# Chromatin-directed proteomics-identified network of endogenous androgen receptor in prostate cancer cells

**DOI:** 10.1038/s41388-021-01887-2

**Published:** 2021-06-14

**Authors:** Kaisa-Mari Launonen, Ville Paakinaho, Gianluca Sigismondo, Marjo Malinen, Reijo Sironen, Jaana M. Hartikainen, Hanna Laakso, Tapio Visakorpi, Jeroen Krijgsveld, Einari A. Niskanen, Jorma J. Palvimo

**Affiliations:** 1 Institute of Biomedicine, Faculty of Health Sciences, University of Eastern Finland, Kuopio, Finland; 2 German Cancer Research Center (DKFZ), Heidelberg, Germany; 3 Department of Environmental and Biological Sciences, University of Eastern Finland, Joensuu, Finland; 4 Institute of Clinical Medicine, Clinical Pathology and Forensic Medicine, University of Eastern Finland, Kuopio, Finland; 5 Department of Clinical Pathology, Kuopio University Hospital, Kuopio, Finland; 6 Faculty of Medicine and Health Technology, Tampere University and Tays Cancer Centre, Tampere University Hospital, Tampere, Finland; 7 Fimlab Laboratories, Tampere, Finland; 8 Heidelberg University, Medical Faculty, Heidelberg, Germany

**Keywords:** Biological sciences, Prostate cancer

## Abstract

Treatment of prostate cancer confronts resistance to androgen receptor (AR)-targeted therapies. AR-associated coregulators and chromatin proteins hold a great potential for novel therapy targets. Here, we employed a powerful chromatin-directed proteomics approach termed ChIP-SICAP to uncover the composition of chromatin protein network, the chromatome, around endogenous AR in castration resistant prostate cancer (CRPC) cells. In addition to several expected AR coregulators, the chromatome contained many nuclear proteins not previously associated with the AR. In the context of androgen signaling in CRPC cells, we further investigated the role of a known AR-associated protein, a chromatin remodeler SMARCA4 and that of SIM2, a transcription factor without a previous association with AR. To understand their role in chromatin accessibility and AR target gene expression, we integrated data from ChIP-seq, RNA-seq, ATAC-seq and functional experiments. Despite the wide co-occurrence of SMARCA4 and AR on chromatin, depletion of SMARCA4 influenced chromatin accessibility and expression of a restricted set of AR target genes, especially those involved in cell morphogenetic changes in epithelial-mesenchymal transition. The depletion also inhibited the CRPC cell growth, validating SMARCA4’s functional role in CRPC cells. Although silencing of SIM2 reduced chromatin accessibility similarly, it affected the expression of a much larger group of androgen-regulated genes, including those involved in cellular responses to external stimuli and steroid hormone stimulus. The silencing also reduced proliferation of CRPC cells and tumor size in chick embryo chorioallantoic membrane assay, further emphasizing the importance of SIM2 in CRPC cells and pointing to the functional relevance of this potential prostate cancer biomarker in CRPC cells. Overall, the chromatome of AR identified in this work is an important resource for the field focusing on this important drug target.

## Introduction

Androgens and androgen receptor (AR), a hormone-activated transcription factor (TF), are key factors driving the development and progression of prostate cancer (PCa). The AR is therefore the primary molecular target for the hormone therapy of advanced PCa [1]. Androgen deprivation therapies, especially with second-generation antiandrogens and androgen synthesis inhibitors are initially effective. However, since patients can still progress from advanced PCa to lethal castration-resistant prostate cancer (CRPC) [2], new therapeutic targets and biomarkers are needed. One source for targets may lie in the AR-associated chromatin proteins.

Most of the currently known nuclear receptor (NR)-interacting proteins, including those of AR, have been identified through genetic screens, such as two-hybrid systems, co-immunoprecipitation, and peptide fragment-based in vitro methods [3–5]. Even though affinity purification-coupled to mass spectrometry (MS) [6, 7] has enlightened the coregulators of NRs, it has rarely been performed in conditions that represent the natural milieu of NRs. Nevertheless, by utilizing RIME (rapid immunoprecipitation MS of endogenous proteins), Paltoglou et al. [8] and Stelloo et al. [9] have identified several endogenous AR-associated proteins from cross-linked chromatin of PCa cells.

Coregulators often reside as subunits in protein complexes and participate in the regulation of transcription in multiple ways, e.g. by modulating histone modifications and chromatin structure. Mammalian BRG1- or BRM-associated chromatin remodeling complex (BAF, SWI/SNF) changes the chromatin accessibility landscapes in cancer cells. The complex is a crucial regulator of cell cycle and proliferation [10], and a driver of PCa [11], with multiple cancer-specific roles [12–14]. Moreover, a frequently occurring TMPRSS2-ERG fusion gene translocation can re-target the BAF complexes on chromatin to promote prostate oncogenesis [15]. Mutually exclusive ATPase subunits, BRG1 (SMARCA4) and BRM (SMARCA2) are the key components for the complex function. In addition, cooperation between AR and DNA sequence-specific TFs, such as FOXA1, GATA2, ERG and HOXB13, is well established [16]. Especially, the pioneer TF FOXA1 can bind to closed chromatin regions to regulate their chromatin accessibility, being able to facilitate chromatin binding of the AR and thus contributing to PCa carcinogenesis [17, 18].

In this work, we utilized chromatin immunoprecipitation coupled with selective isolation of chromatin-associated proteins (ChIP-SICAP) [19] to capture the chromatin protein network, the chromatome (CHROMATin proteOME) of endogenous AR in VCaP cells that represent CRPC cells [20]. In addition to anticipated chromatin remodeling factors, such as SMARCA4, we identified several nuclear proteins not previously associated with the AR. Among the latter were single-minded homolog 2 (SIM2) and aryl hydrocarbon nuclear translocator (ARNT/HIF1β) that together form a heterodimeric TF. SIM2 has intriguingly been reported as a biomarker of aggressive PCa [21, 22], but not previously associated as a partner of AR or with androgen signaling. In addition to identifying the chromatome of endogenous AR in the model CRPC cells, we characterized the genome-wide role of SIM2 in chromatin accessibility, AR binding and gene expression in comparison to that of SMARCA4 in the VCaP cells. Our results uncover interesting gene and pathway-selective roles for both SMARCA4 and SIM2 in the regulation of androgen signaling in CRPC cells.

## Results

### The chromatome of AR in CRPC cells

**Fig. 1:**
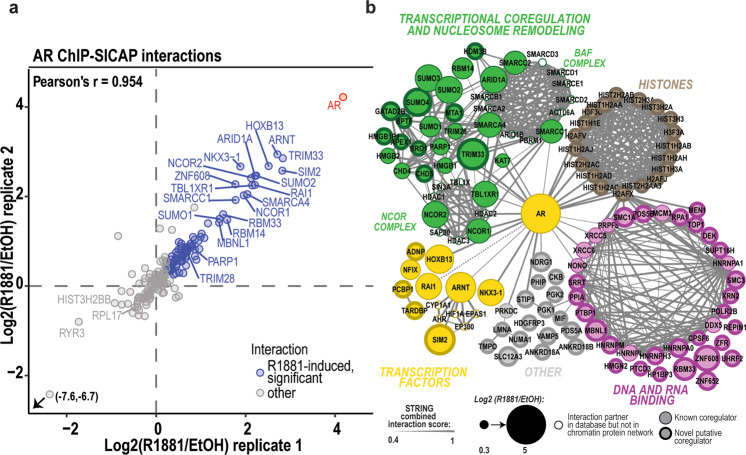
ChIP-SICAP-revealed chromatome of endogenous AR in VCaP prostate cancer cells. **a** Scatter plot showing chromatin-associated proteins identified with AR ChIP-SICAP in two biological replicates in VCaP cells. Significantly R1881-induced (adj. *p* value < 0.05) members of AR chromatome are shown in blue and the AR highlighted in red. Gray dots represent interactions that are not R1881-induced. **b** Members in chromatome of AR grouped and colored by their GO: molecular function annotation. BAF and NCOR complex composition were acquired from CORUM-database and complex members that are not in the chromatome are shown with white filled nodes. Node size represents R1881-dependency (i.e. Log2(R1881/EtOH)) in chromatome and thickness of the edges represents the combined interaction score in STRING-database except for RAI1 whose interaction with AR, described in [23], was not in database. Members of the network not previously linked to AR signaling are highlighted with a thicker border width of the node.

To complement our BioID-derived interactome of AR [23], we used ChIP-SICAP [19] to identify the composition of chromatome around the AR in its chromatin context using differential SILAC-labeling in VCaP cells exposed to R1881 (a synthetic AR agonist), or vehicle (Supplementary materials, Fig. 1a, Supplementary Figure S1a). From the 190 ChIP-SICAP quantified proteins, 87 formed a R1881-induced AR chromatome, thus uncovering proteins that may functionally interact with the receptor (Fig. 1a, Supplementary Fig. S1b, Supplementary Table 1). Overlap between the ChIP-SICAP-identified AR chromatome with two RIME-based interactomes of AR from LNCaP (∼20%) and R1-AD1 PCa cells (∼7%) is shown in Supplementary Fig. S2 [8, 9].

The R1881-induced AR chromatome was categorized to different functional protein groups (Fig. 1b). DNA- and RNA-binding proteins, histones, and DNA repair- and mRNA processing-related proteins formed majority (~50%) of the network. DNA-binding proteins with more specialized roles, e.g. coregulator roles, in transcription formed one-third of the chromatome. This group included ARID1A, SMARCA4, SMARCC1 and SMARCC2, subunits of the BAF complex, and CHD4, a subunit of NuRD complex, coregulators NCOR1, NCOR2 and TRIM28, as well as PARP1, a regulator of DNA damage repair and transcription. An intriguing fraction (~11%) of the chromatome consisted of TFs, e.g. NKX3-1 and HOXB13. Most interestingly, the ChIP-SICAP revealed a large number (>70%) of chromatin proteins that have not previously been identified to associate with AR or androgen signaling (Fig. 1b and Supplementary Table 1). This group includes e.g. MTA1 and GATA2B, two additional subunits of the NuRD complex, BRD1, a component of the MOZ/MORF acetyltransferase complex, and CHD5, an ATP-dependent helicase. TARDBP and SIM2, two TFs and potential PCa biomarkers [21, 22, 24, 25], and prostate metastasis suppressor NDRG1 [26] also belong to the proteins not previously linked to the AR. From the AR chromatome, we characterized and validated the roles of two proteins; SMARCA4 previously linked to the AR and SIM2 hypothesized to act as a novel pioneer TF of AR.

### SMARCA4 co-occupies the majority of AR-binding sites, but has a limited effect on their chromatin accessibility

**Fig. 2:**
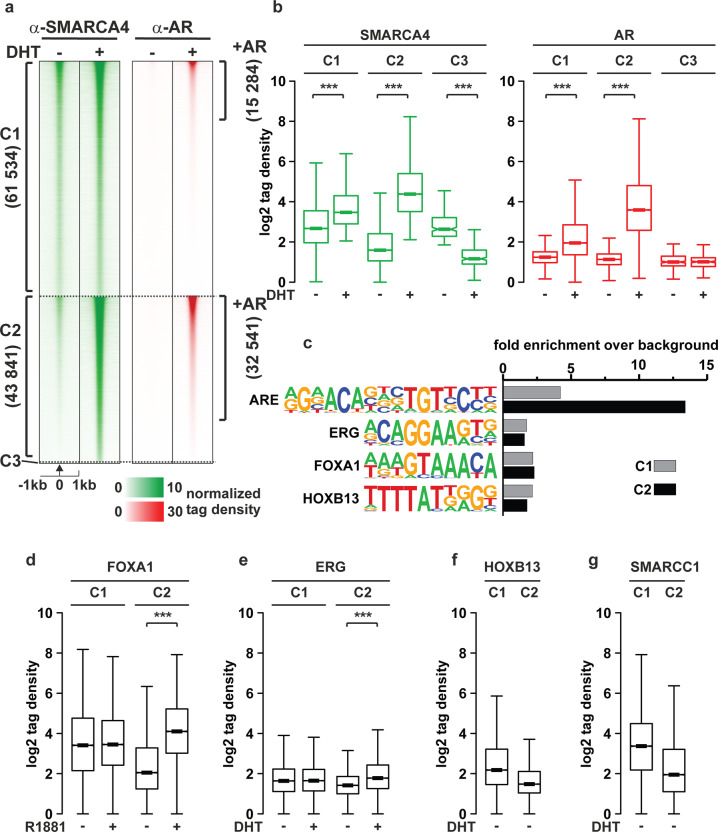
Activated AR recruits SMARCA4 onto chromatin in VCaP cells. **a** Heatmaps of normalized tag counts from ChIP-seq with SMARCA4 and AR antibodies. C1 represents cluster where SMARCA4 is recruited to chromatin independently of DHT exposure (-3 < FC(DHT/vehicle) <3), C2 represents DHT-induced SMARCA4 binding sites (FC(DHT/vehicle)>3) and C3 cluster where SMARCA4 binding is lost by AR activation by DHT (FC(DHT/vehicle) <-3). **b** Boxplots of SMARCA4 (green) and AR tag (red) counts in each cluster in panel **a**. **c** Motif analysis for two first clusters showing the fold enrichment over background**. d**–**f** Binding for FOXA1, ERG or HOXB13 at sites in clusters C1 and C2 in the presence and absence of androgen as indicated. HOXB13 data are from cells grown in normal growth media medium without added androgen. **g** SWI/SNF complex member SMARCC1-binding in sites of clusters C1 and C2. Significant change in binding shown by asterisks ***<0.001, calculated with One-way ANOVA with Bonferroni post hoc test.

To explore the more detailed role of SMARCA4 in AR-associated chromatin environments, we first performed ChIP-seq with SMARCA4 and AR antibodies in VCaP cells in the presence or absence of 5α-dihydrotestosterone (DHT). We focused our interest on sites co-occupied by SMARCA4 and AR and how androgen affects the co-occupancy. Most chromatin-binding sites of SMARCA4 (61534) were not affected by DHT (cluster C1, Fig. 2a, Supplementary Fig. S3a). However, DHT enhances the recruitment of SMARCA4 to these sites (cluster C1, Fig. 2b). The SMARCA4-binding sites (SBs) in C1 prevalently enrich in promoter regions (Supplementary Fig. S3b) marked with active histone modifications and active production of intergenic enhancer RNA (eRNA) without an effect of androgen (Supplementary Fig. S3b, d–f). This suggests that the C1 sites mostly represent VCaP cell type-specific active promoters and enhancers whose activity is mostly unresponsive to androgen. More than 40% of SBs (cluster C2) were induced upon androgen treatment and ~75% of them overlapped with AR-binding sites (ARBs). At the C2 sites, AR and SMARCA4 showed a high correlation with the binding (Fig. 2a and b, cluster C2, Supplementary Fig. S3c) and they enrich in intergenic and intronic enhancer regions with intergenic eRNA production upon hormone exposure (Supplementary Fig. S3b, d–f). Only a few sites were lost upon hormone treatment (cluster C3). Examples of genome browser tracks are shown in Supplementary Fig. S3g–h.

The motif analyses revealed that the C2 sites have a higher enrichment of androgen response elements (AREs) compared with the C1 sites, further supporting androgen-induced recruitment of SMARCA4 onto the chromatin. Motifs for FOXA1, ERG and HOXB13 were in turn equally enriched at the C1 and C2 sites (Fig. 2c, Supplementary Table 2). However, ChIP-seq datasets [27–29] indicated that binding of FOXA1 and ERG increased upon hormone exposure at C2 sites, but not at C1 sites (Fig. 2d and e). HOXB13, however, seems to bind more at C1 sites (Fig. 2f). Similar to SMARCA4, SMARCC1 [15], (found in the ChIP-SICAP), shows binding onto C1 and C2 sites (Fig. 2g, Supplementary Fig. S3d), suggesting that entire BAF-complex is recruited to ARBs.

To gain more insight into how SMARCA4 influences chromatin accessibility at ARBs, we depleted SMARCA4 from VCaP cells (Supplementary Fig. S4) and performed ATAC-seq in the presence and absence of DHT. Interestingly, ATAC-seq analysis revealed that SMARCA4 depletion decreased chromatin accessibility at 8966 sites, while it increased the accessibility at 2038 sites (Supplementary Fig. S5a, b). Motifs for CTCF, FOXA1 and HOXB13 were the most enriched ones at the chromatin sites whose accessibility was decreased by SMARCA4 depletion (Supplementary Fig. S5c). ChIP-seq datasets indicated that FOXA1 and HOXB13 bind more prevalently at the sites whose accessibility was decreased by SMARCA4 depletion than the ones unaffected by the depletion (Supplementary Fig. S5d). These data suggest that SMARCA4 affect the function of FOXA1 and HOXB13.

**Fig. 3:**
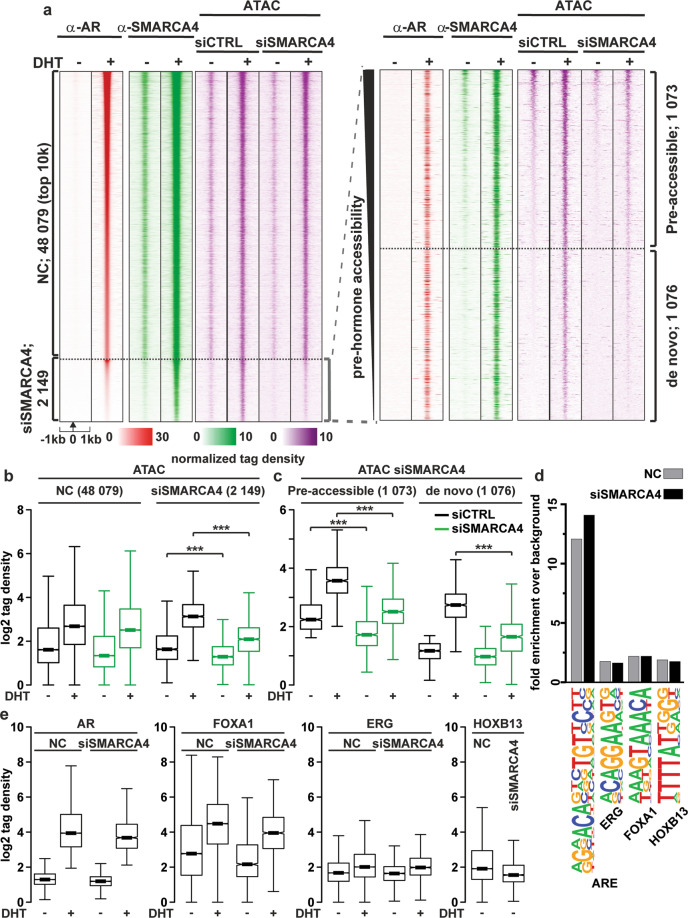
Depletion of SMARCA4 decreases chromatin accessibility at a subset of AR-binding sites as assessed by ATAC-seq. **a** Chromatin accessibility sites as revealed by ATAC-seq in SMARCA4-depleted and control VCaP cells shown as heatmap of normalized tag counts together with SMARCA4 and AR binding. siSMARCA4-affected chromatin accessibility sites magnified and sorted by the pre-hormone (DHT) accessibility on the right. NC, sites not changed by siSMARCA4. **b** Boxplots of site accessibility changes by SMARCA4 depletion compared to control under vehicle or DHT exposure. **c** Boxplots of pre-hormone accessibility sites divided into pre-accessible sites that are open already under vehicle conditions, whereas de novo sites open under DHT exposure**. d** Motif analysis of NC and siSMARCA4-affected sites. **e** Binding of AR, FOXA1, ERG or HOXB13 on the sites in panel **a** in the presence and absence of androgen as indicated. Significant changes in accessibility are shown by asterisks ***<0.001, calculated with One-way ANOVA with Bonferroni post hoc test.

We next focused our analyses on the changes of chromatin accessibility at the ARBs, which revealed that androgen generally increased their accessibility (Fig. 3a, ATAC, siCTRL). Despite a large overlap between SBs and ARBs, chromatin accessibility only at 2149 ARBs was reduced by SMARCA4 depletion (siSMARCA4 in Fig. 3a and b and examples of genome browser tracks in Supplementary Fig. S6a, b). Half of these siSMARCA4-affected sites were open regardless of the hormone exposure (pre-accessible sites) and the rest of them showed an increase in their accessibility after the hormone (de novo sites, Fig. 3a and c). These results suggest that the chromatin remodeling by SMARCA4 at ARBs takes place both at androgen-dependent and independent enhancers.

On the pre-accessible sites, SMARCA4 depletion decreased accessibility independently of DHT, whereas at the de novo sites, the decrease was significant only with DHT. The latter result suggests that the SMARCA4 recruited by the AR increases chromatin accessibility, potentially assisting recruitment of other factors to these sites. This notion was supported by motif analyses (Fig. 3d, Supplementary Table 2). As the binding of especially FOXA1 is androgen-induced at siSMARCA4-affected sites (Fig. 3e), the AR-induced recruitment of SMARCA4 could contribute to the androgen-induced binding of FOXA1 [30]. These results indicate that the SMARCA4 has both AR-dependent and -independent roles in the regulation of chromatin landscape in CRPC cells.

### SMARCA4 modulates the expression of AR target genes involved in extracellular matrix organization and cell adhesion

**Fig. 4:**
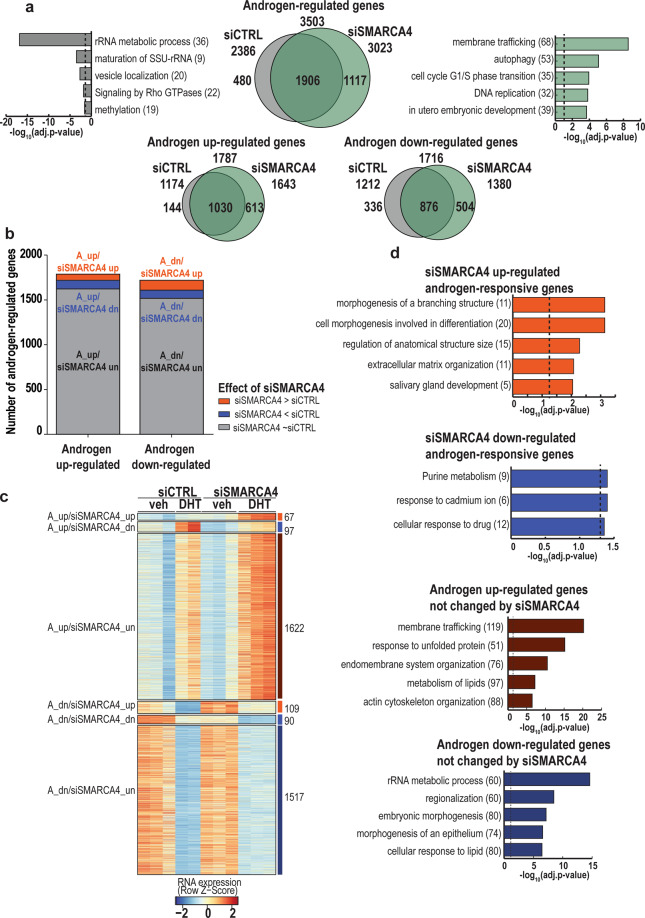
Depletion of SMARCA4 in VCaP cells results in hundreds of differentially expressed androgen-regulated genes as revealed by RNA-seq. **a** Venn-diagrams showing the overlap of androgen-regulated genes (adjusted *p* value < 0.05) in control (siCTRL) and SMARCA4-targeting siRNA (siSMARCA4) -exposed samples. The upper part represents all androgen-regulated genes, with five top enriched pathways for siCTRL unique (in gray) and siSMARCA4 unique genes (in green), and the lower part shows the subdivision of androgen-regulated genes into upregulated and downregulated subpopulations (see also Supplementary Fig. S5). **b** Subdivision of androgen-upregulated and -downregulated genes to six groups as defined by the effect of siSMARCA4. siSMARCA4-upregulated genes (A_up/siSMARCA4_up; A_dn/siSMARCA4_up; adjusted *p* value < 0.05, log2[siSMARCA4 androgen/siCTRL androgen] >0) are shown in red, siSMARCA4-downregulated genes (A_up/siSMARCA4_dn; A_dn/siSMARCA4_dn; adjusted *p* value < 0.05, log2[siSMARCA4 androgen/siCTRL androgen] <0) are in blue, and unchanged genes (A_up/siSMARCA4_un; A_dn/siSMARCA4_un) are in gray. **c** Heatmap showing RNA expression of individual replicates (*n* = 2 for siCTRL DHT and *n* = 3 for others) in the gene groups defined in b. Gene numbers belonging to each group are shown on the right. veh, vehicle. **d** Pathway analysis showing the five most significantly (adjusted *p* value < 0.05, dashed line) enriched pathways in Metascape (see Supplementary Table 3 for details) for the gene groups as defined in panel **b**. Number of genes associated with each biological process is shown in parenthesis.

We next studied the genome-wide effects of SMARCA4 depletion on gene expression in VCaP cells with and without DHT using RNA-seq. Principal component analysis (PCA) showed only small differences upon SMARCA4 depletion, but the effect of DHT remained (Supplementary Fig. S7a). SMARCA4 depletion altered the expression of 1646 genes. As analyzed by Metascape [31], genes that enriched in ribosome biogenesis and translation were the top pathways downregulated by siSMARCA4 with vehicle and DHT, respectively. Mitophagy was in turn the top pathway upregulated by siSMARCA4 with vehicle, whereas the genes upregulated by siSMARCA4 with DHT did not significantly enrich in Metascape analysis (Supplementary Fig. S7b, c). We next focused on androgen-regulated transcriptome; SMARCA4 depletion brought 1117 new genes under androgen regulation, while 480 genes lost their androgen regulation. The majority (~70%) of genes in the latter group were androgen (A) downregulated genes, whereas within the former group, the amount of both upregulated and downregulated genes increased approximately equally (Fig. 4a, Supplementary Fig. S7d). siSMARCA4 resulted in 931 differentially expressed genes (DEGs) compared to siCTRL with DHT, of which 363 genes were significantly androgen-regulated (Fig. 4b, c, Supplementary Fig. S7e). Notably, the expression of 68% of the DEGs that are not regulated by androgen decreased upon SMARCA4 depletion (Supplementary Fig. S7e), clearly indicating that the regulatory role of SMARCA4 in CRPC cells is not restricted to androgen-regulated genes.

Differentially androgen-regulated gene sets were then subjected to Metascape analysis. SMARCA4 depletion increased the expression of androgen-responsive genes enriched in e.g., morphogenesis of a branching structure and cell morphogenesis involved in differentiation, whereas the depletion inhibited the expression of those enriched in purine metabolism and cellular response to a drug (Fig. 4d, see also Supplementary Table 3). From siSMARCA4 DEGs, only A_up/siSMARCA4_dn gene set was associated with siSMARCA4-affected chromatin sites in ATAC-seq data (Supplementary Fig. S8a–c), implying that SMARCA4-mediated changes in chromatin accessibility facilitate their expression.

**Fig. 5:**
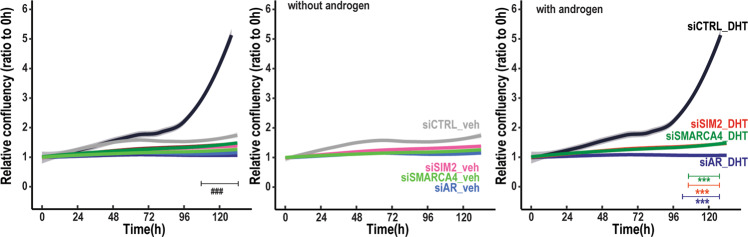
Silencing of SMARCA4 and that of SIM2 attenuates proliferation of VCaP cells. Confluency of VCaP cells exposed to siCTRL, siAR, siSMARCA4 and siSIM2 as indicated was monitored by phase percentage with live cell imaging in Incucyte® in relation to the starting time point. Cells were exposed to 10 nM DHT or vehicle (veh, ethanol) as indicated at the start of the experiment. Line presents mean and the shadow around it indicates standard deviation from four biological replicates. Significant changes to control in Two-way ANOVA and Bonferroni’s multiple comparison posttests are indicated with asterisks, *p* value ***<0.001 and significant hormone effect with ^###^<0.001. On the left, all cell confluence measurements in the same graph; in the middle, measurements in the absence of androgen; on the right, measurements in the presence of DHT.

We next used live-cell imaging that measures cell confluency as a proxy for cell growth and spreading to test whether the effects of SMARCA4 depletion are translated into altered growth of VCaP cells (see Supplementary materials for details). We also compared the effect of SMARCA4 depletion to that of AR depletion [27] (Supplementary Fig. S9). As shown in Fig. 5, SMARCA4 depletion did not influence the cell growth in the absence of androgen, whereas in the presence of androgen, it decreased the relative cell confluency, albeit to a lesser extent than AR depletion. Depletion of SMARCA4 similarly decreased the relative confluency of LNCaP cells (Supplementary Fig. S10), displaying similar expression of SMARCA4 as VCaP cells (Supplementary Fig. S11). Our results thus imply an important role for SMARCA4 in the androgen regulation of genes involved in the extracellular matrix organization and morphogenesis, pathways that include possible connections to epithelial-mesenchymal transition (EMT) in CRPC cells.

### Silencing of SIM2 alters chromatin accessibility at a subset of ARBs

The SIM2, identified here as a novel TF in the AR chromatome, has been reported as a biomarker of PCa [22, 32]. Analysis of a PCa cohort [33] concluded that the expression of SIM2 mRNA is significantly higher in PCa than in benign prostate hyperplasia, with CRPC showing a further increasing trend in the expression (Supplementary Fig. S12). Moreover, high expression of SIM2 mRNA is associated with decreased overall patient survival in TCGA PCa data (Supplementary Fig. S13). To explore the effect of SIM2 on AR-dependent transcription, we first tested if the TF could modulate the activity of AR-dependent reporter gene in VCaP cells (Supplementary Methods). Like SMARCA4, overexpression of SIM2 or its heterodimerization partner ARNT alone did not affect the reporter activity, but increased reporter activity was detected when SIM2 and ARNT were co-transfected (Supplementary Fig. S14a, b), suggesting potential for the SIM2-ARNT heterodimer in the regulation of AR-dependent transcription.

As attempts to study the chromatin binding of endogenous SIM2 using ChIP-seq failed due to lack of suitable antibodies, we silenced SIM2 (silencing confirmed by RT-qPCR, Supplementary Fig. S15) and studied whether this affects accessibility of chromatin at or close to ARBs in VCaP cells. Interestingly, ATAC-seq revealed that SIM2 silencing decreased chromatin accessibility at 10514 sites, while it increased the accessibility only at 351 sites (Supplementary Fig. S16a, b). Motifs for CTCF, FOXA1 and HOXB13 were the most enriched ones at the chromatin sites whose accessibility was decreased by SIM2 silencing (Supplementary Fig.e S16c). Analysis of ChIP-seq data indicated that FOXA1 and HOXB13 bind more prevalently at the sites whose accessibility was decreased by SIM2 silencing than the ones unaffected by the silencing (Supplementary Fig. S16d). These data suggest that similarly to the SMARCA4, the SIM2 might affect the function of FOXA1 and HOXB13.

**Fig. 6:**
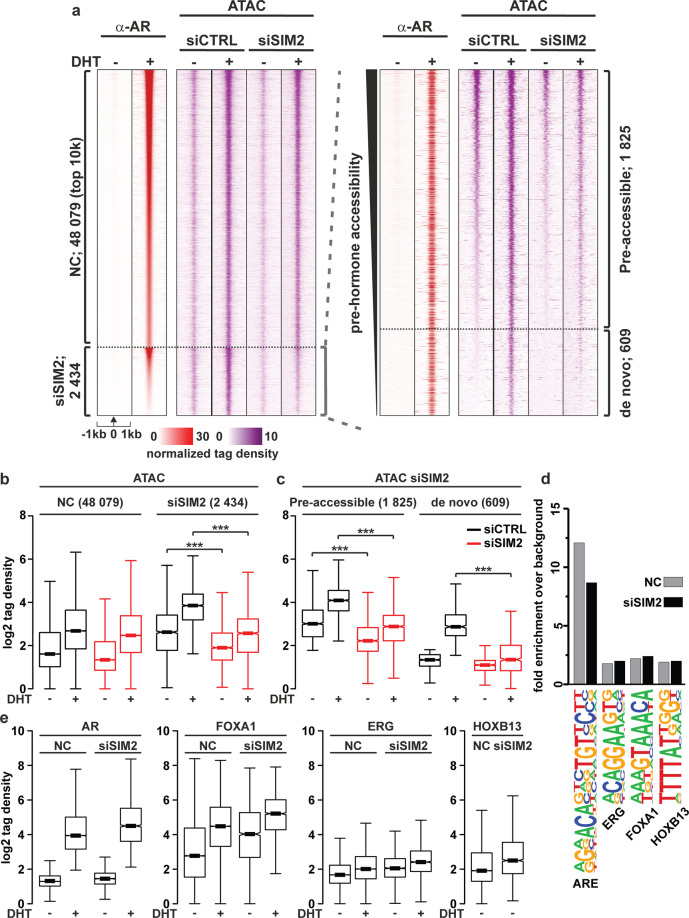
SIM2 binds to pre-accessible chromatin regions and competes or co-operates with binding of other transcription factors. **a** Heatmap of normalized tag counts in AR-ChIP-seq and ATAC-seq from siSIM2 and control experiments in VCaP cells with siSIM2-affected chromatin accessibility sites magnified. **b** Boxplots of ATAC-seq tags in siSIM2-unaffected and -affected chromatin sites (NC and siSIM2, respectively) in the presence and absence of DHT. **c** ATAC-seq tags at siSIM2-affected pre-accessible and de novo sites. Pre-accessible sites are open already without hormone, whereas de novo sites open with DHT. **d** Motif analysis of NC and siSIM2-affected sites. **e** Binding of AR, FOXA1, ERG or HOXB13 at NC and siSIM2-affected sites, respectively in the presence and absence of androgen as indicated. Significant changes in accessibility shown are by asterisks ***<0.001, calculated with One-way ANOVA with Bonferroni post hoc test.

We next focused our analyses on the changes of chromatin accessibility at the ARBs, which revealed that SIM2 silencing reduces the chromatin accessibility at 2434 ARBs (examples of genome browser tracks in Supplementary Fig. S17a–c). Two-thirds of the siSIM2-affected ARBs were accessible before androgen exposure (pre-accessible in Fig. 6a–c), while the rest of them became accessible after androgen exposure (de novo in Fig. 6a–c). In line with the ChIP-SICAP data, the most enriched motif at siSIM2-affected sites was the ARE, albeit it was less enriched at those sites than at the sites unaffected by siSIM2 (NC sites, Fig. 6d, Supplementary Table 2). The tag density of AR, but also that of FOXA1, ERG and HOXB13, showed an increasing trend at the siSIM2-affected sites compared with the NC sites (Fig. 6e). These results suggest that the SIM2 is a TF co-operating with AR and possibly with other PCa-relevant TFs, e.g. FOXA1 or HOXB13.

**Fig. 7:**
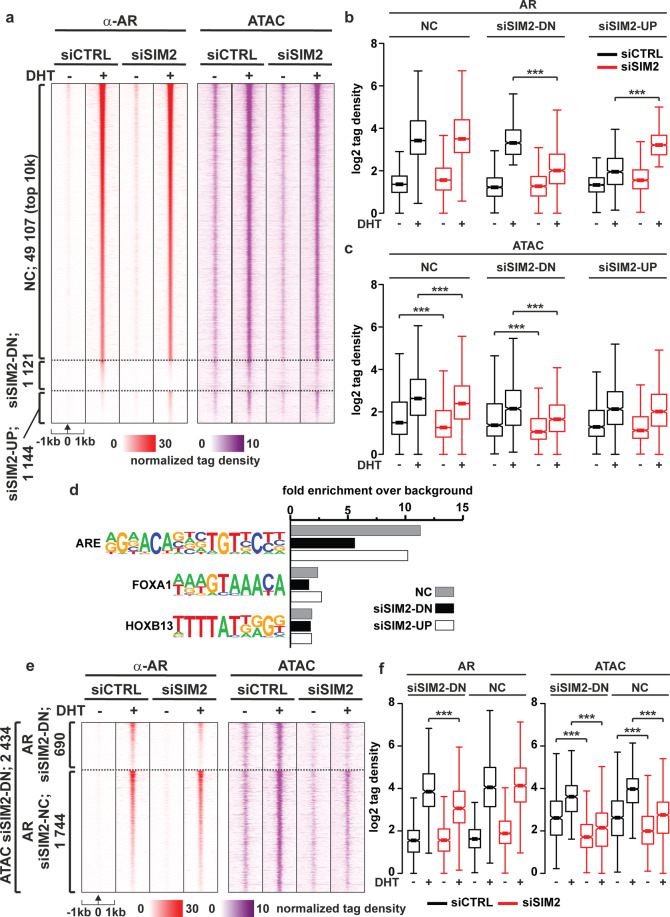
Silencing of SIM2 alters binding of AR to chromatin at more than two thousand chromatin sites. **a** AR-ChIP seq and ATAC-seq data shown as heatmap of normalized tag densities upon SIM2 silencing and vehicle or DHT exposure. Sites where AR binding is decreased (siSIM2-DN) and increased (siSIM2-UP) by siSIM2 are shown in total and together with top10k of non-changed (NC) sites. Boxplot of AR binding (**b**) and chromatin accessibility changes (**c**) in groups defined in panel **a**. **d** Motif analyses of each site group in panel **a**. **e** ATAC-seq results for siSIM2-affected chromatin accessibility sites divided into 690 SIM2- downregulated AR-binding sites and 1744 nonchanged AR-binding sites. **f** Boxplots of **e**. Significance indicated by asterisks ***<0.001 calculated with One-way ANOVA with Bonferroni post hoc test.

To test whether SIM2 also affects the binding of AR to chromatin, we performed AR ChIP-seq after SIM2 silencing. As shown in Fig. 7, binding of the receptor to majority of ARBs was not changed. However, the SIM2 silencing affected 2265 ARBs by reducing and increasing chromatin occupancy at approximately equal number of sites (Fig. 7a, b). When reflecting these to the siSIM2-affected changes in chromatin accessibility, interestingly, a decrease in the accessibility was seen at 690 siSIM2-DN ARBs (Fig. 7c, e, f), whereas at siSIM2-UP ARBs, changes in the chromatin accessibility were not visible (Fig. 7c). The remaining (1744) siSIM2-affected sites showed no change in AR binding. Thus, SIM2 might display some pioneer factor activity with the AR in certain chromatin environments, but its pioneering activity is weaker than that of FOXA1 (Supplementary Fig. S18a–d). Moreover, the majority of ARBs altered by SIM2 silencing did not overlap with ARBs altered by FOXA1 depletion (Supplementary Fig. S18e), which is supported by the motif analyses showing less enrichment of FOXA1 motif at the siSIM2-DN ARBs than at sites showing no change in AR binding (Fig. 7d, Supplementary Table 2). These results together suggest that the SIM2 can render a subset of chromatin sites more accessible to the AR.

### SIM2 has a marked effect on the AR-mediated gene expression

**Fig. 8:**
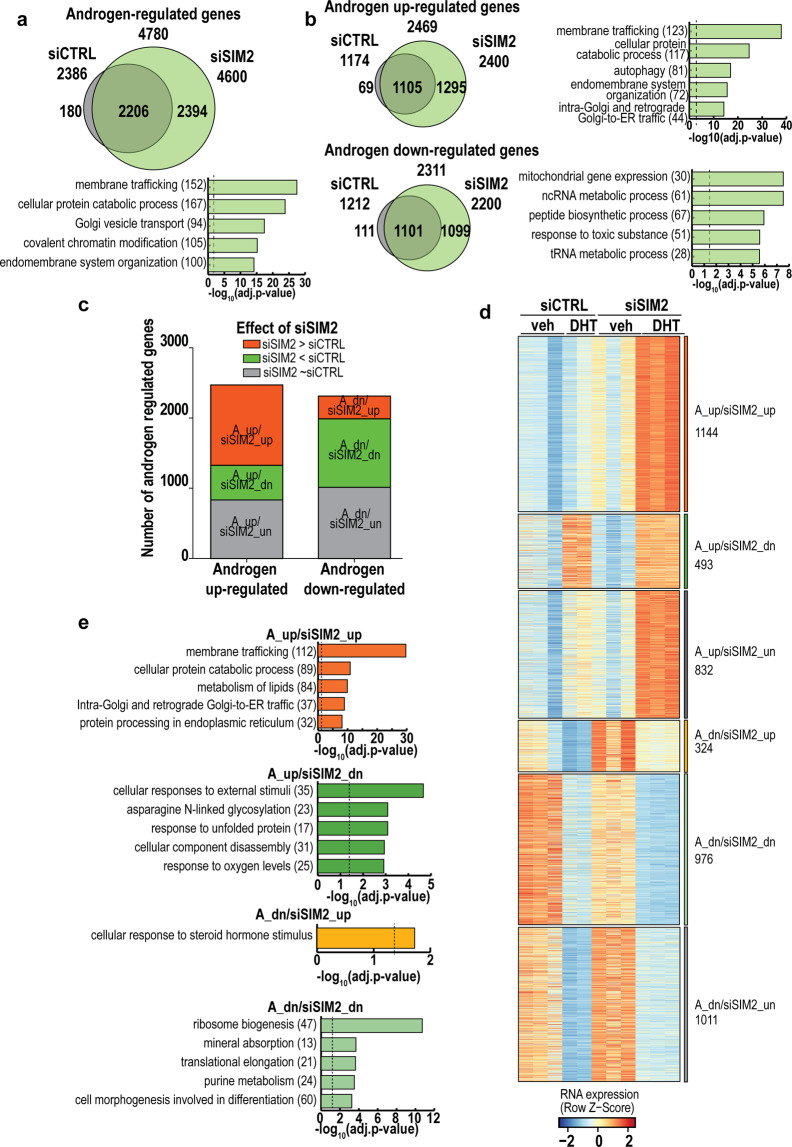
Silencing of SIM2 in VCaP cells yields nearly three thousand differentially expressed androgen-regulated genes as assessed by RNA-seq. **a** Venn-diagrams showing the overlap of androgen-regulated genes (adjusted *p* value < 0.05) in control (siCTRL)- and SIM2-targeting siRNA (siSIM2)-treated cells. Top five enriched pathways for siSIM2 unique androgen-regulated genes are shown in bar plot. **b** Venn diagrams subdividing androgen-regulated genes into upregulated and downregulated subpopulations with their top five enriched pathways in siSIM2 unique genes in bar plots. **c** Subdivision of androgen-upregulated and-downregulated genes to six groups as defined by the effect of siSIM2. siSIM2-upregulated genes (A_up/siSIM2_up; A_dn/siSIM2_up; adjusted *p* value < 0.05, log2[siSIM2 androgen/siCTRL androgen] >0) are shown in red, siSIM2-downregulated genes (A_up/siSIM2_dn; A_dn/siSIM2_dn; adjusted *p* value < 0.05, log2[siSIM2 androgen/siCTRL androgen] <0) in green, and unchanged genes (A_up/siSIM2_un; A_dn/siSIM2_un) in gray. **d** Heatmap showing RNA expression of individual replicates (*n* = 2 for siCTRL DHT and *n* = 3 for others) in the gene groups defined in panel **c**. Gene numbers belonging to each group are shown on the right. veh, vehicle. **e** Pathway analysis showing the five most significantly (adjusted *p* value < 0.05, dashed line) enriched pathway in Metascape for siSIM2 affected gene sets defined in panel **d**, see also Supplementary Fig. S13 and Supplementary Table 3. Number of genes associated with each biological process is shown in parenthesis.

Next, we explored the effect of SIM2 on gene expression using RNA-seq after SIM2 silencing. In PCA, RNA-seq samples formed separate groups, both by siCTRL/siSIM2 and vehicle/DHT treatment (Supplementary Fig. S19a). In comparison to the SMARCA4 depletion, SIM2 silencing had a stronger general effect on gene expression, as the expression of almost 7300 genes was altered (Supplementary Fig. S19b, c). For example, the expression of *BRCA1*, *BRCA2* and *ATM*, genes involved in DNA damage repair, was increased by SIM2 silencing, whereas that of *NSE*, *SYP* and *GHGA*, marker genes of the neuroendocrine PCa, was decreased by the silencing (Supplementary Fig. S19d, e). Metascape showed that cell cycle checkpoints and cell division were the top pathways upregulated by siSIM2 with vehicle and with DHT, respectively (Supplementary Fig. S19b, c, Supplementary Table 3). Interestingly, SIM2 silencing more than doubled the number of DHT-regulated genes, bringing 2394 genes under androgen regulation, while abolishing androgen regulation of only 180 genes (Fig. 8a, b). Furthermore, SIM2 silencing resulted in 6867 DEGs of which 2937 ones were androgen-regulated (Fig. 8c, d, Supplementary Fig. S19f). SIM2 silencing also alleviated the repression of AR expression by androgen (Supplementary Fig. S20). ARNT silencing essentially recapitulated the effect of SIM2 on selected AR target genes and DEGs as assessed by RT-qPCR (Supplementary Fig. S21a, b).

Metascape analysis of androgen-regulated gene sets showed a significant enrichment of several pathways by SIM2 silencing (Supplementary Table 3). The top pathways enriched in the A_up/siSIM2_up gene set and that in the A_up/siSIM2_dn gene set were membrane trafficking and cellular responses to external stimuli, respectively (Fig. 8e, two upper graphs). On the other hand, among the androgen-downregulated genes, siSIM2 enhanced cellular response to steroid hormone stimulus and attenuated ribosome biogenesis (top pathways in A_dn/siSIM2_up and A_dn/siSIM2_dn, respectively, Fig. 8e two lower graphs). In line with the above interpretation of the ATAC-seq data, epigenetic Landscape In Silico deletion Analysis [34] revealed enrichment of FOXA1 with the DEGs, especially among the siSIM2-upregulated genes (see Supplementary Table 4).

From the DEGs, siSIM2-upregulated genes (A_up/siSIM2_up and A_dn/siSIM2_up) were associated with siSIM2-affected sites in ATAC-seq data (Supplementary Fig. S22a), as exemplified by *CA13* locus (Supplementary Fig. S22b) and *UGT8* locus (Supplementary Fig. S22c). To summarize these genome-wide results, SIM2 affects chromatin accessibility at ARBs, albeit to a markedly smaller extent than FOXA1. In comparison to SMARCA4, the relatively small effect of SIM2 on chromatin accessibility of ARBs is translated to a more pronounced impact on the regulation of gene expression, affecting interesting regulatory pathways.

### Proliferation of CRPC cells and their tumor size are repressed by SIM2 silencing

Since pathway analysis predicted that cell cycle checkpoints and division are affected by SIM2 silencing, we hypothesized that SIM2 silencing affects the cell proliferation. We therefore monitored the effect of SIM2 silencing on VCaP cells with live-cell imaging and compared effect of the silencing to that of AR depletion. As assessed by relative cell confluence, SIM2 silencing decelerated VCaP cell growth similarly as that of AR or SMARCA4 (Fig. 5). Proliferation of androgen-exposed LNCaP cells was not however attenuated by SIM2 silencing (Supplementary Fig. S10), but without androgen, SIM2-silenced LNCaP cells interestingly proliferated faster than their controls. The reason for these differences between these PCa lines may derive from ∼20-fold higher expression of SIM2 mRNA in VCaP cells than LNCaP cells (Supplementary Fig. S11). To complement the above data with VCaP cells in three-dimensional tumor growth, we used chick embryo chorioallantoic membrane (CAM) assay (images of representative *in ovo* tumors in Supplementary Fig. S23a). In line with the results from the cell culture experiments, SIM2 silencing similarly to AR depletion decreased tumor size of VCaP cells in CAM assays (Supplementary Fig. S23b), which further points to the importance of SIM2 in the regulation of CRPC cells.

## Discussion

Here, we utilized ChIP-SICAP which purifies exclusively chromatin-bound functional interactors [19] to cast new light to the protein associations and function of AR. Our ChIP-SICAP quantified proteins binding to AR-containing enhancers in PCa cell milieu. Other proteomic approaches that have captured proteins in the proximity of AR on chromatin in PCa cells utilized RIME in CWRR1-derived R1-AD1 cells and LNCaP cells [8, 9]. Although these studies reported similar numbers of proteins as our ChIP-SICAP in VCaP cells, the overlap of identified proteins is small, probably reflecting the intrinsic differences between the methodologies and cell line models. Only three BAF complex members, ARID1A, SMARCA4 and SMARCC1, are identified in all three studies. Both RIME and ChIP-SICAP combine ChIP of endogenous protein complexes with MS analysis, however in ChIP-SICAP, ChIP is followed by a purification step to specifically capture DNA-bound proteins [19, 35]. Therefore, the investigation on chromatin-bound functional interactors of AR through ChIP-SICAP in VCaP cells resulted in a smaller number of identified proteins than RIME in LNCaP cells (190 *vs*. 333), but a larger portion of the ChIP-SICAP quantified proteins was significantly enriched with AR in androgen-dependent fashion (46% vs. 20%, respectively). VCaP cells, but not LNCaP cells, additionally express an AR variant, AR-V7, devoid of the ligand-binding domain (LBD) [36]. Moreover, the VCaP cells, but not the LNCaP cells, express a TMPRSS2-ERG fusion, and the AR-LBD in LNCaP is point-mutated. These differences may contribute to the low overlap between the AR chromatin protein interactomes of the VCaP and the LNCaP cells, but they are not likely to explain all of them.

SMARCA4 is an established chromatin remodeler [37] and one of the shared members of AR chromatomes identified in three studied PCa cell lines [8, 9]. It is overexpressed in TCGA PCa data [38] and PCa cell lines [39]. Higher SMARCA4 expression correlates with aggressiveness of the disease, but irrespective of clinical or molecular subtype [12, 13, 39, 40]. SMARCA4 and PTEN (frequently lost in PCa) are interestingly synthetic lethal in PCa [13], which is reflected in PCa cell models as an altered chromatin structure driving a protumorigenic transcriptome [13].

To the best of our knowledge, the impact of androgen-activated AR on the chromatin occupancy of SMARCA4, the chromatin accessibility and the AR target gene expression has not previously been investigated in an integrative fashion in PCa cells. Our data from VCaP cells show that despite a marked number of SMARCA4 chromatin-binding sites responded to androgen (>40%) and a large overlap (75%) between SMARCA4- and AR-binding sites, the depletion of SMARCA4 changed the chromatin accessibility <10% of those sites. This relatively small effect of SMARCA4 depletion may be explained by two interchangeable ATPase subunits, SMARCA4 and SMARCA2, the latter of which was not however detected in our AR ChIP-SICAP. Moreover, our data did not indicate any compensatory increase in the expression of SMARCA2 upon SMARCA4 depletion. Since enhancers recruit remodelers with different affinity [41], removal of SMARCA4 and BAF activity could have paved the way to another remodeling activity to the enhancer. The NuRD complex whose subunits, MTA1, GATA2B and CHD4 (ATPase subunit), identified here could represent an alternative remodeling activity. In fact, many enhancers require the activity of two or more remodeling complexes, e.g. SMARCA4 and CHD4-containing ones [41].

Regardless of the relatively limited effect of SMARCA4 on the chromatin accessibility on ARBs, SMARCA4 depletion affected chromatin accessibility at a larger number of sites (~11000). The depletion did not only decrease the accessibility, but the accessibility at ~20% of the SMARCA4 depletion-affected sites was increased, with FOXA1 being enriched at decreased sites. The FOXA1 has been assumed to create open chromatin environment by displacing linker histones [42, 43]. Our results suggest that BAF complexes might have more prevalent roles in the FOXA1-mediated regulation of chromatin accessibility than previously postulated.

The SMARCA4 depletion in VCaP cells had interesting effects on AR-mediated gene expression, as e.g. the expression of androgen-regulated genes involved in the extracellular matrix organization and morphogenesis of branching structures were sensitive to SMARCA4. These pathways include possible connections to EMT that has previously been indicated as a cellular process regulated by SMARCA4 [44–49]. Similarly to our study, larger and more general effects in chromatin accessibility than those in gene expression were demonstrated by SMARCA4 in other cancer cell models [50, 51]. We found that SMARCA4 depletion practically blunted the proliferation-promoting effect of androgen in VCaP cells, which is accordance with others’ studies with LNCaP cells in which the effect of androgen was not however addressed [39, 40].

Our chromatin-directed proteomics approach revealed SIM2, a basic helix-loop-helix Per-Arnt-Sim (bHLH-PAS) TF [52], and its heterodimerization partner ARNT as members of AR chromatome. The bHLH-PAS TFs play important roles in morphogenesis and controlling circadian rhythmicity, responses to hypoxia and toxin metabolism [53]. The SIM2-ARNT heterodimer can either activate or repress transcription depending on the gene context [52, 54, 55]. This notion is in line with our genome-wide data from PCa cells, showing that silencing of SIM2 influenced - activated and repressed - similar numbers of androgen-regulated target genes. For example, the expression of genes enriched in cellular response to steroid hormone stimulus were enhanced by SIM2 silencing, whereas the expression of genes enriched in ribosome biogenesis were repressed by the silencing. Additionally, SIM2 silencing showed androgen-independent effects on genes involved in cell cycle process, cell division and DNA repair; e.g. enhancing the expression of *BRCA1*, *BRCA2* and *ATM*. These roles are in line with SIM2’s growth-promoting role [56–58], ability to regulate DNA damage repair [59, 60] and increase invasion potential [61]. Moreover, we show that the proliferation of VCaP cells and their tumor size are affected by SIM2 expression. Since the SIM2 is overexpressed in PCa, with the expression increasing with the aggressiveness of PCa [21, 22], these data point to the relevance and importance of SIM2 as a PCa biomarker. Moreover, given the role of SIM2 in neurogenesis [62, 63] and that the expression of neuroendocrine markers *SYP*, *CHGA* and *NSE* [64, 65] are attenuated by SIM2 silencing, the SIM2 may also have a role in the neuroendocrine PCa.

Our ATAC-seq showed that, although most of the ARBs were unaffected by SIM2 silencing, the accessibility at >2000 ARBs was altered. Chromatin at most of the affected sites was accessible before androgen exposure, suggesting that SIM2 preferentially binds to and acts at open chromatin regions. In addition, binding of AR was affected - attenuated or enhanced - at ~2000 sites by SIM2 silencing. However, there was no simple relationship between the changes in chromatin accessibility and the AR binding upon SIM2 silencing. SIM2 could display some pioneer factor properties with AR in certain chromatin environments, but these properties are much weaker than those of the FOXA1. Moreover, the majority of ARBs affected by SIM2 silencing do not overlap with those altered by FOXA1 depletion. Together, our results indicate that the SIM2 is a TF co-operating with AR in CRPC cells.

In conclusion, our results confirm the chromatin opening role of SMARCA4 in AR-mediated gene regulation, which is interestingly reflected in the expression of genes involved in pathways potentially connected with EMT in PCa. In addition, our results indicate that SIM2 plays an important, AR target pathway-selective role in the regulation of CRPC cells. Finally, the chromatome of AR in CRPC cells identified herein forms an important resource for the AR field, focusing on this important drug target.

## Materials and methods

### Cell culture

VCaP cells were obtained from ATCC, tested negative for mycoplasma, and verified by Institute for Molecular Medicine Finland. Cells were maintained in DMEM (Gibco #41965-039) supplemented with 10% FBS (HyClone #AYE161472) and 1 U/µl penicillin and 1 µg/ml streptomycin (Gibco #15140-122).

### ChIP-SICAP

SILAC-labeled VCaP cells (9 × 10^6^ cells/ 15-cm culture dish, Supplementary materials) were cultured for 72 h, before adding 10 nM R1881 to heavy-labeled (Arg10, Lys8) cells and ethanol (vehicle) to light-labeled (Arg0, Lys0) cells for 1 h. Cells were immunoprecipitated as in [66] with following modifications; the chromatin was incubated overnight with αAR [67] followed by 3 h incubation with rotating G protein-coupled DynaBeads (Invitrogen) at 4 °C. ChIP protocol was subsequently followed by [19, 35]. (Details and data analysis in Supplementary materials).

### RNAi followed by RNA-sequencing

For RNA-sequencing, VCaP cells (0.7 × 10^6^ cells/well of 6-well plate) were reverse-transfected with ON-TARGETplus SMARTpools for SMARCA4, SIM2 and non-targeting control (Dharmacon, Supplementary materials) using RNAiMAX (ThermoFisher Scientific) according to the manufacturer’s instructions (Details in Supplementary materials).

### ChIP- and ATAC-sequencing

VCaP cells (5 × 10^6^ cells/10-cm culture dish) were cultured in maintenance medium for 48 h. Medium was changed to DMEM, 2.5% charcoal-stripped FBS for 48 h prior to 1 h exposure with DHT 100 nM or vehicle. In siRNA-experiments, SMARCA4, SIM2 or FOXA1 were silenced by reverse transfection with ON-TARGETplus SMARTpools for 96 h prior hormone treatment. ChIPs were performed as in [66] with αSMARCA4 (Abcam, #ab110641), αFOXA1 (Abcam, #ab23738) and αAR [67]. The libraries were prepared with NEB Next^®^Ultra DNA™ II kit (E7103, NEB) and sequenced with Illumina NextSeq 500 (75SE). (Details and data analysis in Supplementary materials).

## Supplementary information

Supplementary materials

Supplementary Table 1

Supplementary Table 2

Supplementary Table 3

Supplementary Table 4

## Data Availability

The MS data have been deposited to the ProteomeXchange Consortium via the PRIDE [79] partner repository with the dataset identifier PXD025193. ATAC-seq, ChIP-seq and RNA-seq datasets have been submitted to GEO database with accession code: GSE136016.
