# Supplementary material for: Chromatin-directed proteomics-identified network of endogenous androgen receptor in prostate cancer cells
Source: Oncogene. 2021 Jun 14;40(27):4567–79. doi: 10.1038/s41388-021-01887-2 (PMC8266679; doi:10.1038/s41388-021-01887-2)
Supplement: Supplementary file 1 — Supplementary materials [file 41388_2021_1887_MOESM1_ESM.pdf]

Supplementary materials
for

Chromatin-directed proteomics-identified network of endogenous androgen receptor in prostate cancer cells

Kaisa-Mari Launonen, Ville Paakinaho, Gianluca Sigismondo, Marjo Malinen, Reijo Sironen, Jaana M. Hartikainen, Hanna Laakso, Tapio Visakorpi, Jeroen Krijgsveld, Einari A. Niskanen, Jorma J. Palvimo

Supplementary Methods

Stable isotope labeling by amino acids in cell culture (SILAC)

VCaP cells were divided into SILAC medium (DMEM, high glucose, no glutamine, no lysine, no arginine, Thermo Fisher #A1443101) supplemented with 10% dialyzed FBS (Thermo Fisher #26400044), 2 mM L-glutamine (Gibco, #25030), 1 U/µl penicillin, 1 µg/ml streptomycin,). The medium was sterile filtered with 0.2 µm filter. Arginine and lysine (from Sigma-Aldrich) were added to final concentrations of 84 µg/ml and 146 µg/ml, respectively. For light-labeling L-arginine (#A8094) and L-lysine monohydrochloride (#L8662) and for heavy-labeling L-arginine-$^{13}C_6$,$^{15}N_4$ hydrochloride (#608033) and L-lysine-$^{13}C_6$,$^{15}N_2$ hydrochloride (#608041) were used. VCaP were maintained in SILAC-mediums for at least ten doubling cycles before SICAP experiments to ensure the incorporation of labeled amino acids into proteins.

Details for ChIP-SICAP

SILAC-labeled VCaP cells were divided 9×10^6 cells on 15-cm culture dish and cultured 72 h. For the last hour 10 nM R1881 was added to heavy-labeled cells and ethanol as a vehicle control for light-labeled cells. SILAC-labeled VCaP were immpunoprecipitated as described in [1] with following modifications; the chromatin was incubated overnight with αAR [2] followed by 3 h incubation with G-protein coupled magnetic DynaBeads (Invitrogen) rotating in 4 °C. ChIP protocol was then followed by SICAP protocol from [3, 4]. Briefly, 3'-ends of sheared DNA were in vitro biotinylated through terminal deoxynucleotidyl transferase (ThermoFisher Scientific EP0161) and used as bait in streptavidin pull-down with protease-resistant streptavidin beads (prS, ref[4]). Beads were then conditioned in 50mM NH4HCO3. Samples were subjected to reduction with 7mM DTT (final concentration) at 55°C for 30min, followed by alkylation with 12mM iodoacetamide at 22°C for 40 min in the dark. Reaction was quenched with DTT and proteins were digested on prS beads with a Trypsin/LysC mix (Promega, V5071) at 37°C for 16 h. Digested peptides were desalted with 2 µl of SP3 para-magnetic beads as previously described [3-6]. Peptides were eluted in 0.1% trifluoroacetic acid (TFA) in H2O, loaded on a trap column (PepMap100 C18 Nano-Trap 100µm x 2cm) and separated over a 25 cm analytical column (Waters nanoEase BEH, 75 µm x 250 mm, C18, 1.7 µm, 130 Å) using the Thermo Easy nLC 1200 nanospray source (Thermo EasynLC 1200, Thermo Fisher Scientific). Solvent A was water with 0.1% formic acid and solvent B was 80% acetonitrile, 0.1% formic acid. During the elution step, the percentage of solvent B increased in a linear fashion from 3% to 8% in 4 min, then increased to 10% in 2 min, to 32% in 68 min, to 50% in 12 min and finally to 100% 1 min and went down to 3% for the last 11 minutes. Peptides were analyzed on a Tri-Hybrid Orbitrap Fusion mass spectrometer (Thermo Fisher Scientific) operated in positive (+2kV) data dependent acquisition mode with HCD fragmentation. The MS1 and MS2 scans were acquired in the Orbitrap and ion trap, respectively with a total cycle time of 3sec. MS1 detection occurred at 120000 resolution, AGC target 1E6, maximal injection time 50 ms and a scan range of 375-1500 m/z. Peptides with charge states 2 to 4 were selected for fragmentation with an exclusion duration of 40s. MS2 occurred with CE 33%, detection in topN mode and scan rate was set to Rapid. AGC target was 1E4 and maximal injection time allowed of 50 ms. Data were recorded in centroid mode.

Reporter gene assay

Expression vectors for human SIM2 and ARNT (HIF1β) were obtained from Addgene. SIM2-pDest was a gift from Roger Reeves (Addgene plasmid #53790; [7]) and pCDNA3.1-Flag-HIF1β(#930) gift from James Brugarolas (Addgene plasmid #99916; [8]). The pHaloTag-SMARCA4 was purchased from Promega (pFN21AE0798). Reporter gene analysis in VCaP cells was performed as described in [9] with 600 ng pGL3-ARE2-TATA-LUC, 100 ng pCMVβ (Clontech) and 150 ng SMARCA4, SIM2 or ARNT expression vector. The total amount of DNA per well was balanced to 1 µg with pFLAG-CMV-2. SIM2 and ARNT were also transfected together. Twenty-four h after transfection cells were exposed to 100 nM DHT or vehicle (ethanol) for 17h and luciferase activity, β-galactosidase activity and protein levels were measured as described in [10].

ATAC-sequencing

VCaP cells (5×10^6 cells on 10 cm dish) were reverse transfected with 30 nM ON-TARGETplus SMARTpools for siSMARCA4, siSIM2 and non-targeting control using RNAiMAX (ThermoFisher Scientific) according to the manufacturer's instructions in maintenance media without penicillin and streptomycin. 48 h after transfection the media was changed to assay media for another 48 h. Cells were exposed to vehicle, 100 nM DHT for 1 h. Subsequently, ATAC-seq samples was prepared as described [11, 12] and transposed DNA was amplified with PCR using primers published in [13]. The transposed DNA was mixed with Universal PCR primer 1 (final conc. 1.25 µM), barcoded PCR primer 2 (final conc. 1.25 µM) and 1x NEBNext High-Fidelity 2× PCR Master Mix in total volume of 50 µl. Amplification was done by incubating samples first 5min in 75°C followed by 30s in 98°C. This primary extension cycle was followed by five cycles of denaturation (10 s, 98°C), primer annealing (30 s, 63°C), and extension (1 min, 72°C). Further amplification was checked by qPCR and taking 10 % of sample and mixed it with 1.56 µM primers and qPCR SYBR green master mix (Roche) in total volume of 20 µl. Additional cycles were counted to reach 1/3 of the maximum signal. Samples from two biological replicates were sequenced with Illumina NextSeq 500 (75PE).

ChIP-sequencing

VCaP cells were divided 5×10^6 cells on 10-cm culture dishes in maintenance medium for 48 h. Medium was changed to assay medium (2.5 % charcoal-stripped FBS in DMEM) 48 h prior to 1 h exposure with DHT 100 nM, or vehicle (EtOH). In siRNA-experiments, cells were reverse transfected with 30 nM ON-TARGETplus SMARTpools for SIM2 or 60 nM ON-TARGETplus SMARTpools for FOXA1 (Dharmacon, Table S1) and using RNAiMAX transfection reagent according to the manufacturer's instructions in maintenance medium without penicillin and streptomycin 96 h prior hormone treatments and 48h prior medium change. The cistromes were produced as described in [1] with αSMARCA4 (Abcam, #ab110641), αFOXA1 (Abcam, #ab23738) and αAR [2] and the libraries were prepared with NEB Next®Ultra DNA™ II lib preparation kit (E7103, NEB) and with Illumina NextSeq 500 (75SE).

RNA interference followed by RNA-seq or RT-qPCR

For RNA-sequencing, VCaP cells (0.7×10^6 cells/well on 6-well plates) were reverse-transfected with 30 nM ON-TARGETplus SMARTpools for SMARCA4 and SIM2 or non-targeting control (Dharmacon, Table S1) using RNAiMAX (ThermoFisher Scientific) according to the manufacturer's instructions in maintenance media without penicillin and streptomycin. After 48 h, the medium was changed to assay media (DMEM, 2.5 % charcoal-stripped FBS) for 24 h. Cells were exposed to 100 nM DHT or vehicle (ethanol) for 6 h. RNA was extracted with RNeasy® Plus Mini Kit (Qiagen) and mRNA isolated using NEBNext Poly(A) mRNA Magnetic Isolation Module (E7490, New England Biolabs) according to manufacturer's recommendations. RNA-seq libraries were prepared using NEBNext Ultra II Directional RNA Library Prep Kit (E7765, New England Biolabs) and pooled libraries were sequenced with Illumina NextSeq 500 (75SE).

For RT-qPCR VCaP cells (0.7×10^6 cells/well on 6-well plates) were reverse-transfected with 30 nM ON-TARGETplus SMARTpools for SMARCA4, SIM2, ARNT or non-targeting control (Table S1) using RNAiMAX (ThermoFisher Scientific) according to the manufacturer's instructions in maintenance media without penicillin and streptomycin. After 48 h the medium was changed to assay media (DMEM, 2.5% charcoal-stripped FBS) for 24 h. Cells were exposed to 100 nM DHT or vehicle (ethanol) for 6 h. RNA was extracted with TriPure (Roche) according to the manufacturer's instructions. Extracted RNA was used as a template in two-step reverse transcriptase-qPCR (RT-qPCR) by converting it first to cDNA using Transcriptor First Strand cDNA Synthesis Kit (Roche). cDNA was used as a template in RT-qPCR as previously described [1] with primers listed in Table S2.

Immunoblotting

Cell monolayers from silencing experiments in VCaP cells were washed with ice-cold PBS and collected in PBS complemented with 1x protease inhibitor cocktail (PIC, Roche) and 10mM NEM. Cell pellets were suspended in SDS-PAGE sample buffer complemented with 1x PIC and 10 mM NEM and heated at 95 °C for 5 min. Samples were sonicated 2 × 10 s and β-mercaptoethanol was added to final concentration of 5%. Samples were re-heated for 5 min at 95°C before separated on 7.5% SDS-PAGE gels. Proteins were transferred onto nitrocellulose membranes (0.45μm, Thermo Scientific) and visualized by antibodies against SMARCA4 (Abcam, ab110641), FOXA1 (Abcam, ab23738), AR [2], GAPDH (Santa Cruz, sc-25778), or LaminB1 (Santa Cruz, sc-6216). Appropriate horseradish peroxidase-conjugated secondary antibodies using the enhanced chemiluminescence detection reagents according to the manufacturer's instructions (Pierce) were used.

Table S1. *Details of siRNAs for RNAi*

siSIM2	ON-TARGETplus Human SIM2 (6493) siRNA-SMARTpool,<br>Cat no. L-008598-00-0010<br>Dharmacon ON-TARGETplus SMARTpools for RNAi
siARNT	ON-TARGETplus Human ARNT (405) siRNA-SMARTpool,<br>Cat no. L-007207-00-0010<br>Dharmacon ON-TARGETplus SMARTpools for RNAi
siSMARCA4	ON-TARGETplus Human SMARCA4 (6597) siRNA-SMARTpool,<br>Cat no. L-010431-00-0020<br>Dharmacon ON-TARGETplus SMARTpools for RNAi
siFOXA1	ON-TARGETplus Human FOXA1 (3169) siRNA-SMARTpool,<br>Cat no. L-010319-00-0020<br>Dharmacon ON-TARGETplus SMARTpools for RNAi
siCTRL	ON-TARGETplus Non-targeting Pool<br>Cat no. D-00181-10-50<br>Dharmacon ON-TARGETplus SMARTpools for RNAi
Custom siRNA targeting AR	target: 5'-AAGGAGTTGTGTAAGGCAGTG-3'<br>sense: r(GGA GUU GUG UAA GGC AGU G)dTdT<br>antisense: r(CAC UGC CUU ACA CAA CUC C)dTdT<br>Qiagen

Table S2. *RT-qPCR Primers for AR-target genes and siSIM2 DEGs*

GENE	FORWARD	REVERSE
**GAPDH**	TGGGGAAGGTGAAGGTCGG	TCTCAGCCTTGACGGTGCC
**RPL13A**	ACGACAAGAAAAAGCGGATGG	AGGGCAACAATGGAGGAAGG
**SIM2**	GCAGACTTTGGATGGATTTG	TCATCTCATCGTGGTCAGAA
**SMARCA4**	TGCTGCTGCCCCCACGAG	CAATGGTCGCTTTGGTTCGC
**AR**	TTGGAGACTGCCAGGGAC	TCAGGGGCGAAGTAGAGC
**PSA**	GGCAGGTGCTTGTGGCCTCTC	CACCCGAGCAGGTGCTTTTGC
**FKBP5**	AAAAGGCCAAGGAGCACAAC	TTGAGGAGGGGCCGAGTTC
**S100P**	ATGACGGAACTAGAGACAGCC	AGGAAGCCTGGTAGCTCCTT
**SLC45A3**	GGCGGAACCAGCCTGCAC	CTGCTTCGTCTCGGCTCTG
**CLDN8**	CGTGAGGCAGGCTAACATCA	AGCAGCACACATCAGTCCTC
**CDKN1A**	GCAGACCAGCATGACAGATTT	GGATTAGGGCTTCCTCTTGGA
**TMPRSS2**	CCTCTGGTCACTTCGAAG	GTAAAACGACGTCAAGGACG
**TP53**	ATCTACAAGCAGTCACAGCAC	GCTCATAGGGCACCACCAC
**FOSL2**	ACCATCAACGCCATCACGAC	ACGCTTCTCCTCCTCTTCAG
**IGFBP5**	GAAGGACCGCAGAAAGAAGC	GTCCACGCACCAGCAGATG
**ARNT**	TGATGATGGAGAAGGGAACA	TCTCTCTTTATCCGCAGAGC
**SOX9**	GTACCCGCACTTGCACAAC	TCTCGCTCTCGTTCAGAAGTC
**ITGAV**	AGGTCCTCAAATGTGACTGG	TCTCGCTCCTGTTTCATCTC
**TMTC1**	GAGAGTCCCTATTCAGGTCTGG	TGTCTCTCGTCAGTGTTCCA
**WASF3**	GCGGACCGTTTTAGTTTTG	CGTATGATAGCGGCAAGAGT

Cell proliferation monitoring by live cell imaging

VCaP cells were divided 0.5×106 cells/well on 6-well plates in normal growth media 48 h before siRNA transfection. Media was changed to assay media (DMEM, 2.5% charcoal stripped FBS) and incubated 3 h before transfection with siRNAs (40 nM, Table S1). siAR (Table S1) was used as a positive control since its known effect on PCa cell proliferation [8]. After 72 h, cells were seeded onto 96-well ImageLockTM plate (Sartorius, #4379, 20 000 cells/well) in assay media in four biological replicates. Six hours later cells were exposed to 10 nM DHT or vehicle (ethanol). Cells were then monitored every 4 h with automated cell-imaging in IncuCyte® for five and half days and analyzed using parameters listed in Table S3. Data was normalized to starting point and changes during the follow-up are shown as relative changes. Significance differences to control were calculated in GraphPad Prism 8 with Two-way ANOVA and Tukey's multiple comparison posttests comparing all columns. Data was then visualized in R-studio version 1.2.5033 and R v3.6.0, utilizing ggplot package and Adobe Illustrator 2020 version 24.1.1.

Table S3. Parameters for live cell imaging analysis

Cell confluence (phase-contrast imaging)

**masking**	minimum area filter	50 μm^2
	segmentation adjustment	0.7
	clean up size	50 μm^2

Chick embryo chorioallantoic membrane (CAM) assay

VCaP cells were reverse transfected with 30 nM siRNAs for SIM2 (Dharmagon), AR (Qiagen) or non-targeting control (Dharmagon) in growth medium without penicillin and streptomycin five days before introducing cells onto eggs. CAM assay was conducted as previously described [14], except 2×106 cells per egg (siAR, siSIM2 or control silenced VCaP) were applied, and on EDD12 tumors were photographed *in ovo* and embryos sacrificed. Tumor size was evaluated from 9 or 10 eggs per silencing condition by measuring the tumor area from photographs with Image J 1.47v [15] and normalized to plastic ring area by dividing the areas in Microsoft Excel. Data was illustrated by GraphPad Prism 8.4.0 and Adobe Illustrator 2020 version 24.1.1.

LNCaP cells experiments

Lymph Node Carcinoma of the Prostate (LNCaP) cells were obtained from ATCC. Their identity was verified by Institute for Molecular Medicine Finland (FIMM, Helsinki) and they were routinely checked to be negative for mycoplasma. LNCaP cells were maintained in RPMI-1640 (Gibco, 41965-039) supplemented with 10 % FBS (Gibco, 41965-039) and 1U/µl penicillin, 1µg/ml streptomycin (Gibco15140-122) and 2 mM L-glutamine (Gibco, 25030-024). For automated monitoring of cell proliferation, LNCaP cells (15 000 cells/well) were reverse-transfected in four biological replicates with 20 nM SMARCA4- or SIM2-targeting siRNAs (Table S1) or non-targeting siRNAs (siCTRL) with Lipofectamine RNAiMAX transfection reagent (Invitrogen) on 96-well plates in regular growth medium without penicillin and streptomycin. After 36 h, medium was changed to steroid-depleted medium. Four hours later, quadruplicates of cells were exposed to 100 nM DHT or vehicle. Proliferation assay was performed as described for VCaP cells.

Data analysis

ChIP-SICAP

RAW MS data were analyzed with MaxQuant version 1.6.2.6 based on the Andromeda search [16, 17] and peptide identification was performed using Uniprot database of Human (canonical, 2016 release with 20187 entries). Protein and peptide FDRs were set to 1 %. Methionine oxidation and N-terminal acetylation were set as variable modifications, while carbamidomethylation of cysteine residues was set as fixed modification. Match between runs, second peptide and re-quantify options were active and both Label Free Quantification (LFQ) and intensity-Based Absolute Quantification (iBAQ) scores were calculated. The mass spectrometry proteomics data have been deposited to the ProteomeXchange Consortium via the PRIDE [18] partner repository with the dataset identifier PXD025193. Only proteins with at least 1 unique peptide in both replicates of AR pulldown were used for further processing steps. Additionally, common contaminants such as keratins were removed before further processing. R1881-induced chromatome of AR was discriminated from total ChIP-SICAP data with moderated t-statistics with linear model analysis (limma [19]) in R by using adjusted p-value < 0.05 and LogFC (R1881/EtOH) > 0 as cut-off values. Data was illustrated with ggplot in R-studio version 1.2.5033 and R v3.6.0, and Adobe Illustrator 2020 version 24.1.1. Only a master protein of MS identified protein groups was used for visualization purposes. The complete list of R1881-induced interactors and MS group composition is reported in Supplementary Table 1.

ChIP-seq and ATAC-seq

ChIP-seq data analysis was performed as previously described [1, 9]. For ATAC-seq data, after filtering low quality reads as with ChIP-seq data analysis, paired-end samples were aligned to hg38 genome using Bowtie2 [20]. Alignment was performed with end-to-end sensitive mode allowing no mismatches. Around 10-20 % of the reads were mapped to mitochondrial DNA. From the two biological replicate sample, at least 40 million unique non-mitochondrial reads were obtained for each condition. Downstream data analysis was performed using HOMER [21]. Peaks in each data-set were called using findPeaks with style factor, FDR<0.01, >25 tags, > 4-fold over control sample and local background. IgG sample from VCaP cells was used as control sample for ATAC-seq and SMARCA4 ChIP-seq data. EtOH treated AR sample from VCaP cells was used as control for AR ChIP-seq data. Altered binding populations between EtOH and DHT -treated cells for SMARCA4 ChIP peaks, or between siCTRL and siSMARCA4/siSIM2 -treated cells for ATAC peaks were defined with getDifferentialPeaks. Differential peaks had 3-fold difference between treatments and Poisson p-value less than 0.0001. Peaks that showed no difference between treatments were classi-fied as non-changed (NC) sites. Pre-accessible and de novo sites were defined on the basis of ATAC-seq data. De novo sites had <1.5 log2 tags, while pre-accessible sites had > 1.5 log2 tags in EtOH-treated ATAC-seq sample from siCTRL treated VCaP cells. Aggregate plots and heatmaps were generated with 10 bp or 20 bp bins surrounding ±1 kb area around the center of the peak. All plots were normalized to 10 million mapped reads and further to local tag density, tags per bp per site. Box plots and scatter plots represented log2 tag counts. Pearson correlation coefficiency (PCC) is calculated in the scatter plots. Annotatepeaks.pl was used to calculate the enrichment of sites to different genomic location. De novo motif searches were performed using findMotifsGenome.pl with the following parameters: 200 bp peak size window, strings with two mismatches, binomial distribution to score motif p-values, and 50 000 background regions Motif data was displayed as fold enrichment of the motif at target sites compared to background regions (Supplementary Table S2). Statistical significance in the box plots was determined with One-way ANOVA with Bonfer-roni post hoc test. Published datasets were analyzed as indicated above.

Association of differentially regulated genes to ATAC peaks was done with AnnotatePeaks.pl. For each cluster of differentially regulated genes, the fraction of genes with ATAC peak within 100 kb of TSS was calculated. Subsequently, these fractions were compared to control cluster of andro-gen-regulated genes; androgen up- or down-regulated genes with no change with siSMARCA4 or siSIM2. Statistical significance in the association comparisons was done with X2-test.

RNA-seq

In house R pipeline was used to process the sequencing data. Sequenced raw reads were quality controlled, and adapters and reads smaller than 36 nt were removed. Trimmed raw reads were mapped against to Homo sapiens (human) genome assembly GRCh38 (hg38) from Genome Reference Consortium using STAR [22]. One replicate from siCTRL DHT was removed from further analysis due to the considerable difference to all other samples. Total count per gene was calculated using TPM normalization. Differentially expressed genes were analyzed with DESeq2 at HOMER [21] for all comparisons. Other than protein coding genes were filtered out as outliers. Genes with TPM >0.5 at least in one sample in any treatment were considered as expressed and those with adjusted p-value <0.05 as differentially expressed. Genes were grouped to androgen downregulated (adj. p-value<0.05 and log2(FC) < 0) and androgen upregulated (adj. p-value<0.05 and log2(FC) > 0) within each DHT to vehicle comparison. Genes were further defined to upregulated, downregulated (adj. p-value<0.05 and log2(FC)> 0) or unchanged by specific siRNA in DHT conditions. Differentially expressed gene sets were subjected to pathway analysis in Metascape with default settings [23]. Data was visualized with R-studio version 1.2.5033 and R v3.6.0, and Adobe Illustrator 2020 version 24.1.1.

Statistical analyses

Experiments were conducted with at least two biological replicates. Number of biological replicates in each experiment are indicated in methods, figures and/or figure legends. Sample size was determined based on previous studies, literature references, and pilot experiments. Statistical tests used in the study are commonly used and considered appropriate for the hypotheses tested and indicated in respective figure legends. The data meet assumptions of population distribution. Variance between the groups that are being statistically compared is similar. Differences were considered significant at $p < 0.05$. Data are presented as mean ± standard deviation.

Public datasets

The following publicly available sequencing datasets were used: ChIP-seq data for SMARCC1 (GSE110657,ref.[24]), H3K27ac (ENCSR597ULV, ENCODE, ref.[25]), CTCF (ENCSR265ARE, ENCODE, ref.[25]), H3K4me2 (GSE56086,ref.[9]), H2A.Zac (GSE76336, ref.[26]), FOXA1 (GSE56086, ref.[9]), ERG (GSE49091,ref.[27]) and HOXB13 (GSE96652, ref.[28]), and GRO-seq (GSE84432, ref.[29]).

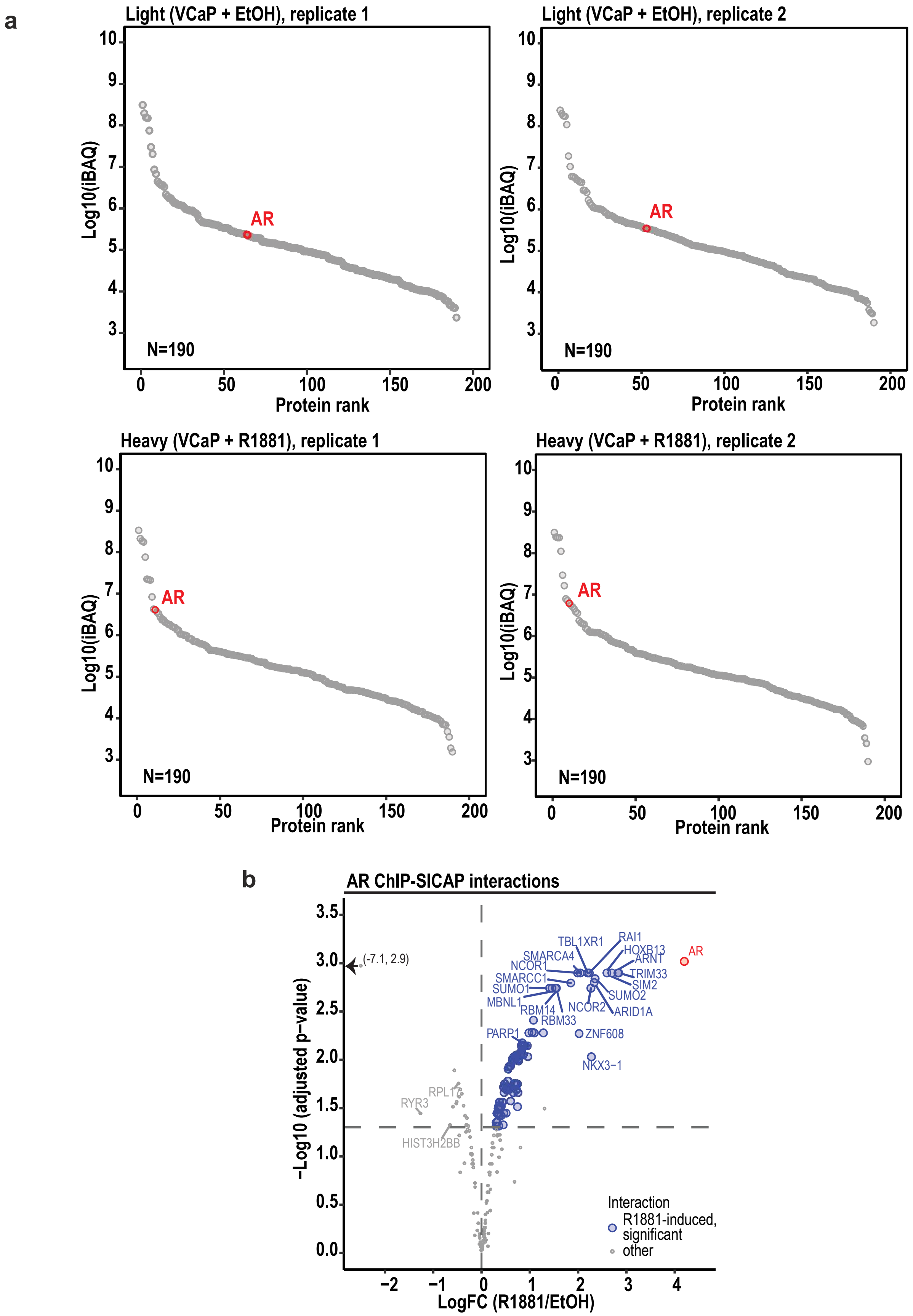

Supplementary Figure S1. Validation of AR ChIP-SICAP analyses. a Ranked iBAQ values for 190 proteins that had >1 identified peptide in AR pulldowns with heavy SILAC- or light SILAC-labeled replicate 1 and replicate 2 showing the signal intensities. **b** Volcano plot shows chromatin-associated proteins identified with AR ChIP-SICAP. Significantly (adj. p-value < 0.05) R1881-induced interactors are shown in blue. Coloring is the same as in Figure 1.

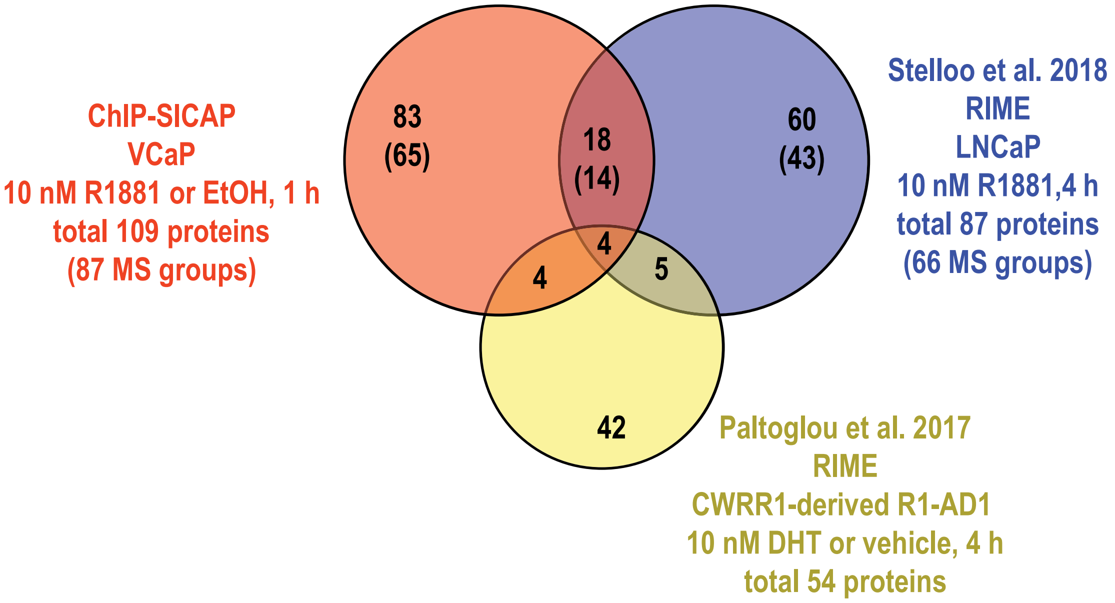

Supplementary Figure S2. Overlap of ChIP-SICAP-identified chromatome of AR from VCaP cells with RIME-identified chromatomes of AR from LNCaP and R1-AD1 cells. Venn diagram comparing two published AR chromatomes by Stelloo et al. 2018 (blue, [30]) and Paltoglou et al. 2017 (yellow, [31]), respectively, with the AR chromatome identified in this work (red). Method for identification, cell line and androgen concentration with exposure time are shown. Total protein numbers refer to all identified proteins. However in mass spectrometry results, the identified peptide may have matched to a protein group (such as different subtypes of histones) rather than individual proteins. The number of MS protein groups are shown in brackets.

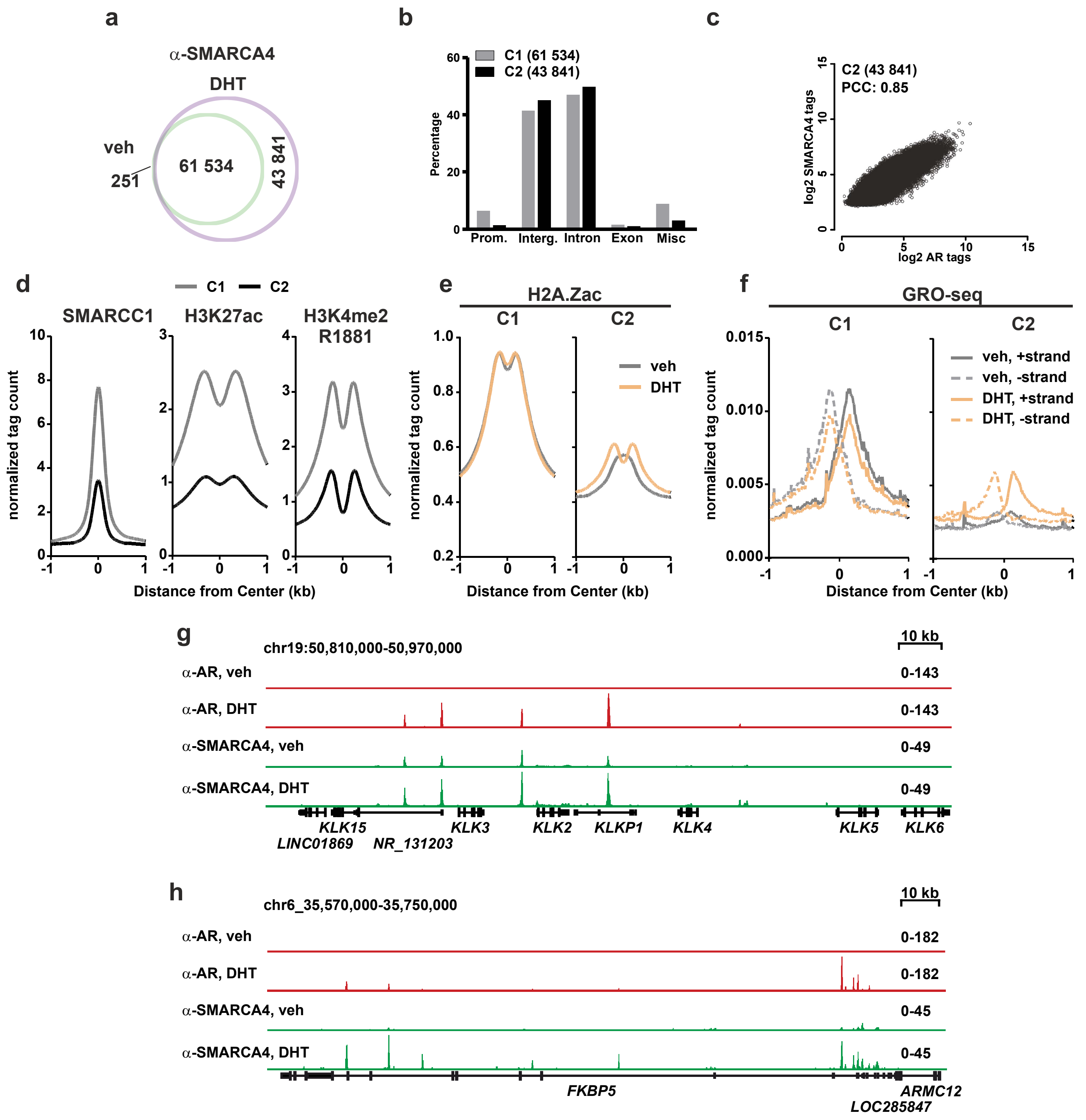

Supplementary Figure S3. Characteristics of SMARCA4-occupied chromatin regions in VCaP cells. **a** Venn-diagram shows anti-SMARCA4 ChIP-seq-identified sites in the presence of vehicle (EtOH) or DHT: 251 sites were lost upon DHT treatment, 61 534 sites did not change, and 43 841 sites were induced by DHT. **b** Division of non-changed (C1 in Figure 2) and DHT-induced SMARCA4-binding sites (C2 in Figure 2) on promoters, intergenic, intronic, exon and miscellaneous genomic regions**. c** Correlation plot of AR- and SMARCA4-binding at sites in C2 shows a high correlation (PCC, Pearson correlation coefficient). **d** ChIP-seq tag counts of another SWI/SNF complex member SMARCC1 and active histone modifications (H3K27ac and H3K4me2) at C1 and C2 sites **e** ChIP-seq tag counts of H2A.Zac at C1 and C2 sites in each treatment. **f** GRO-seq tag counts of eRNA production in both strands and in each treatment at C1 and C2 sites reveal that DHT-induced SMARCA4 sites are actively transcribed. **g** and **h** Examples of genome browser tracks AR target genes where SMARCA4 binds before activation of AR (*KLK* locus) or where activation of AR recruits SMARCA4 onto chromatin (*FKBP5* locus).

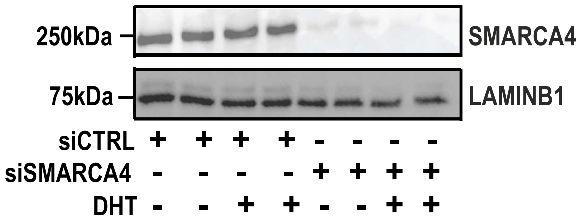

Supplementary Figure S4. Depletion of SMARCA4 in VCaP cells was verified by immunoblotting. Two biological replicates of siCTRL- or siSMARCA4-exposed cells in the presence and absence of DHT (16 h) were analyzed by immunoblotting with anti-SMARCA4 antibody. Anti-LaminB1 antibody was used as a loading control.

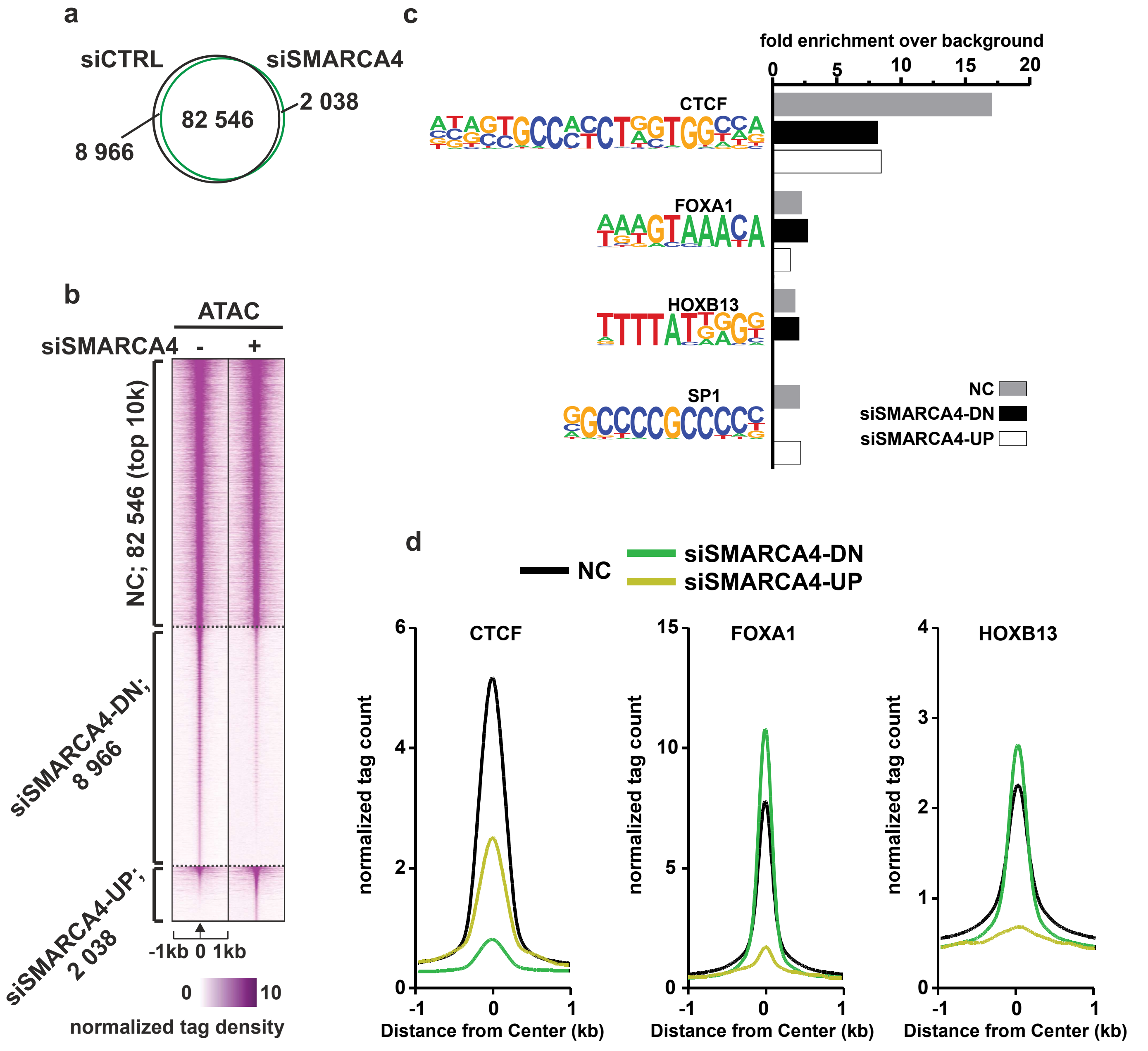

Supplementary Figure S5. Effect of SMARCA4 depletion on overall chromatin accessibility in VCaP cells a and **b** Chromatin accessibility sites as revealed by ATAC-seq in SMARCA4-depleted (siSMARCA4) and control (siCTRL) VCaP cells shown as Venn diagram (**a**) and as a heatmap of normalized tag counts (**b**). SMACRA4 depletion decreased chromatin accessibility at 8 966 sites, whereas at 2 038 sites, the chromatin accessibility was increased. Only top 10k of all chromatin accessibility sites that do not change by SMARCA4 are shown in heatmap (NC). **c** Motif analysis of siSMARCA4-regulated chromatin accessibility sites revealed enrichment of motifs for CTCF, FOXA1, HOXB13 and SP1. For comparison, the enrichment of these motifs is shown for non-changed sites as well. **d** Binding of CTCF, FOXA1 or HOXB13 at NC and siSMARCA4-affected sites.

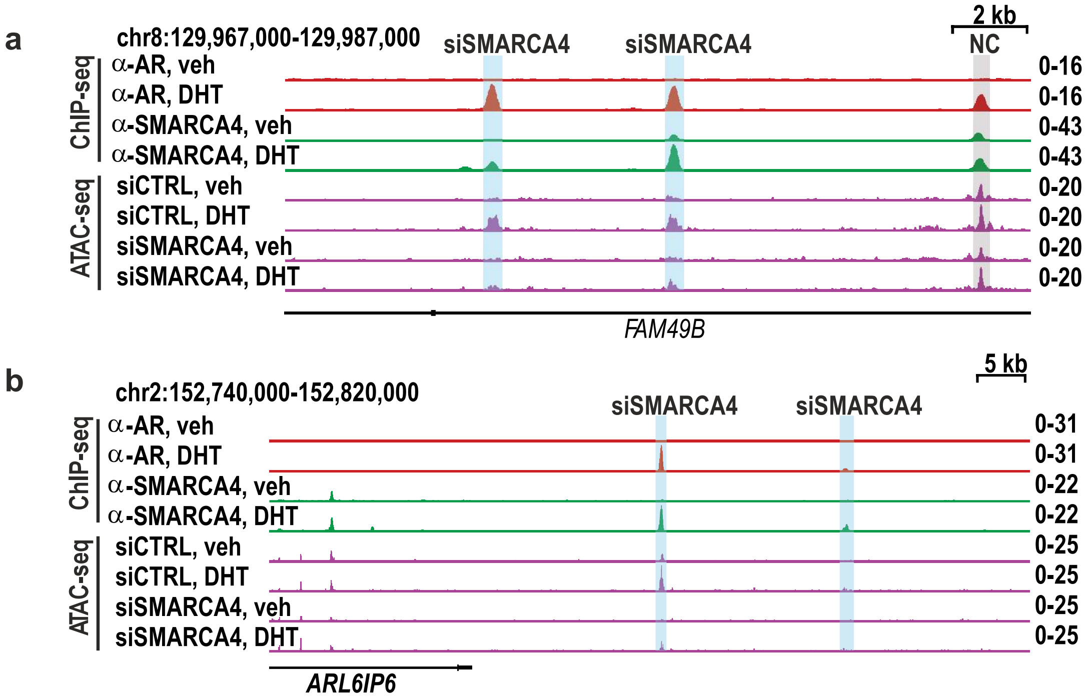

Supplementary Figure S6. Effect of SMARCA4 depletion on chromatin accessibility and of expression selected AR loci in VCaP cells. a and **b.** Examples of genome browser tracks, *FAM49B* and *ARL6IP6* locus, show a decrease in chromatin accessibility upon SMARCA4 depletion at sites co-occupied by SMARCA4 and AR. On the right, scale of peak intensity for each track**.**

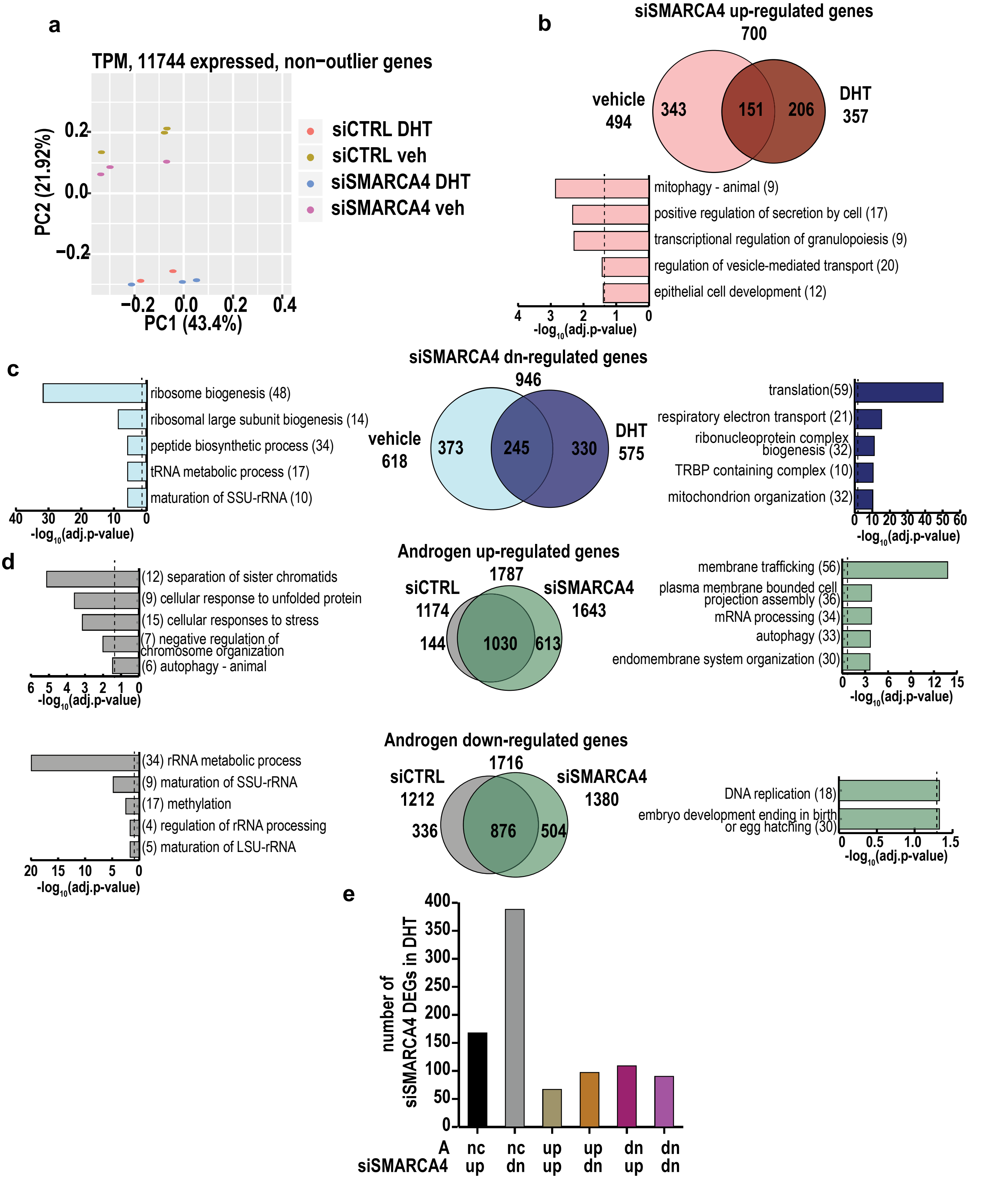

Supplementary Figure S7. Effect of SMARCA4 depletion on gene expression in VCaP as assessed by RNA-seq. a Principal component analysis of expressed genes (TPM>0.5). PC1 differentiates samples by treatment (vehicle, veh; androgen, DHT) and PC2 by siRNA (siCTRL, siSMARCA4). **b** Venn diagram shows overlap of siSMARCA4-up-regulated genes in vehicle or DHT treatment and bar graph shows top five enriched pathways in Metascape analysis within the group of siSMARCA4-up-regulated genes with vehicle (343 genes, light red) , whereas the group with DHT (206 genes) does not enrich significantly in any pathway. **c** Venn diagram of siSMARCA4-down-regulated genes with vehicle or DHT treatment with top five enriched pathways in the presence of vehicle (373 genes, light blue, left) or DHT (330 genes, dark blue, right). **d** Subdivision of androgen-regulated genes into up-regulated and down-regulated groups in Venn diagrams in the middle, and five top enriched pathways for unique genes either in siCTRL (grey, left) or in siSMARCA4 treatment (green, right). **e** Bar graph showing the portions of all differentially regulated genes, and classification of the genes by the effect (nc, not changed; up, up-regulated; dn, down-regulated) of siSMARCA4 and androgen (A). Dashed line in the pathway bar graphs indicates the adjusted p-value 0.05.

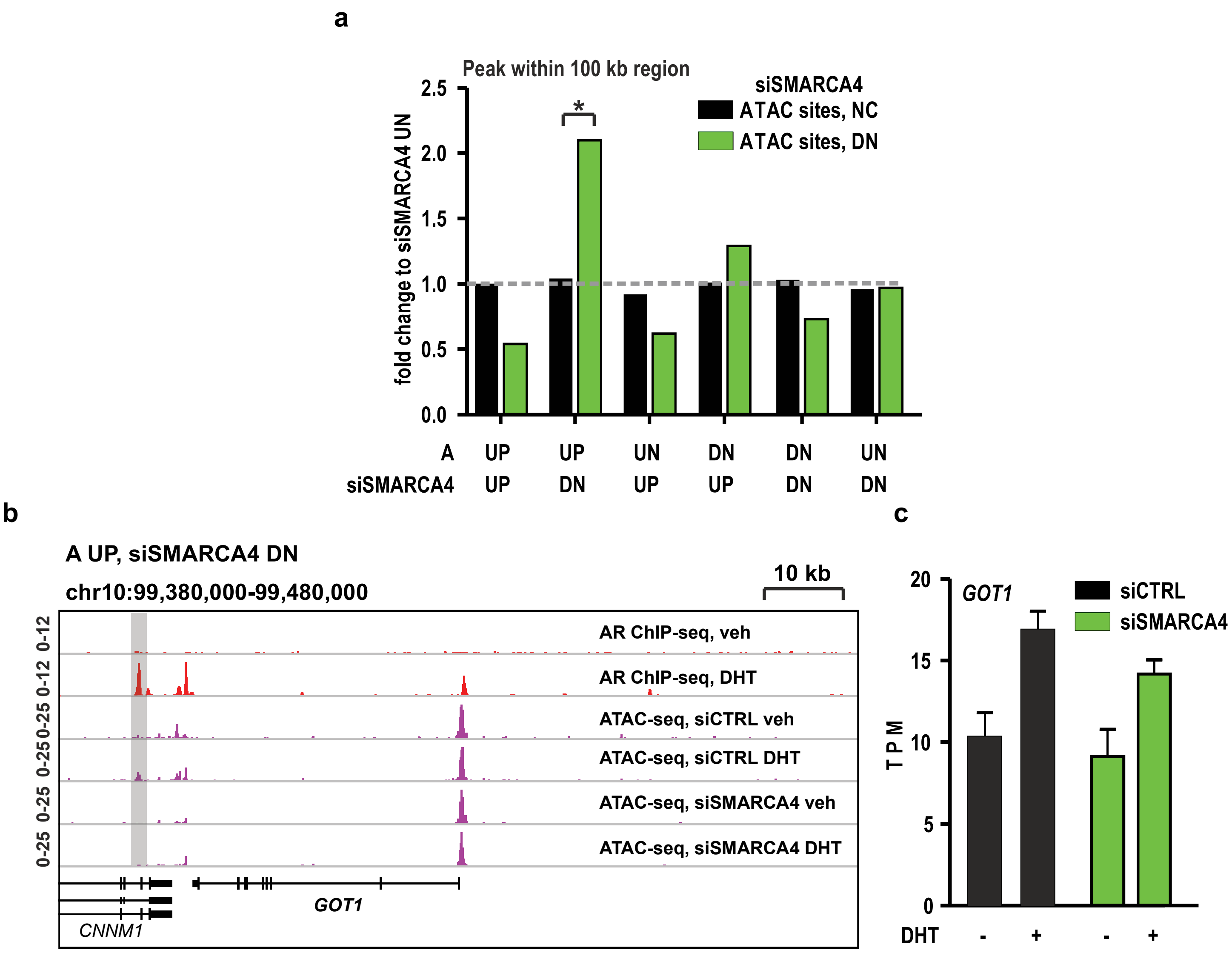

Supplementary Figure S8. siSMARCA4-suppressed and androgen-up-regulated DEGs enrich close to siSMARCA4-affected ATAC-seq sites. a Association of DEGs in siSMARCA4-treated cells (Figure 4, Supplementary Figure S6) with ATAC-seq peaks (Figure 3) with the search criteria of peak within 100 kb from transcription starting site and normalization to androgen-regulated genes whose expression does not change with siSMARCA4 upon DHT exposure. Androgen up-regulated and siSMARCA4-down-regulated (A_up/siSMARCA4_dn) genes were significantly enriched close to siSMARCA4-affected ATAC-seq sites compared to A_up/siSMARCA4_un genes. **b** and **c** *GOT1* locus, as an example, harbors a siSMARCA4-affected site near to its 3'-end and its androgen-regulated expression is decreased upon SMARCA4 depletion. Columns presents mean ± standard deviation from three biological replicates.

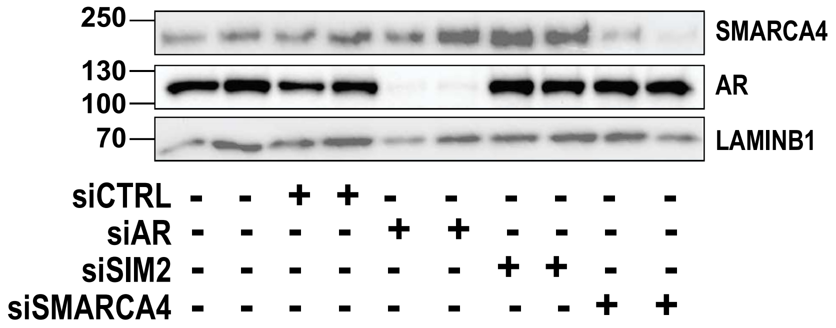

Supplementary Figure S9. Verification of SMARCA4 and AR depletion in VCaP cells by immunoblotting. Two biological replicates of cells without siRNA transfection or transfected with specific siRNAs were analyzed by immunoblotting with anti-SMARCA4 and anti-AR antibodies. Due to the lack of suitable antibody against SIM2 for immunoblotting, silencing of SIM2 was verified by RT-qPCR (see Supplementary Figure S15). Anti-LaminB1 antibody was used as a loading control.

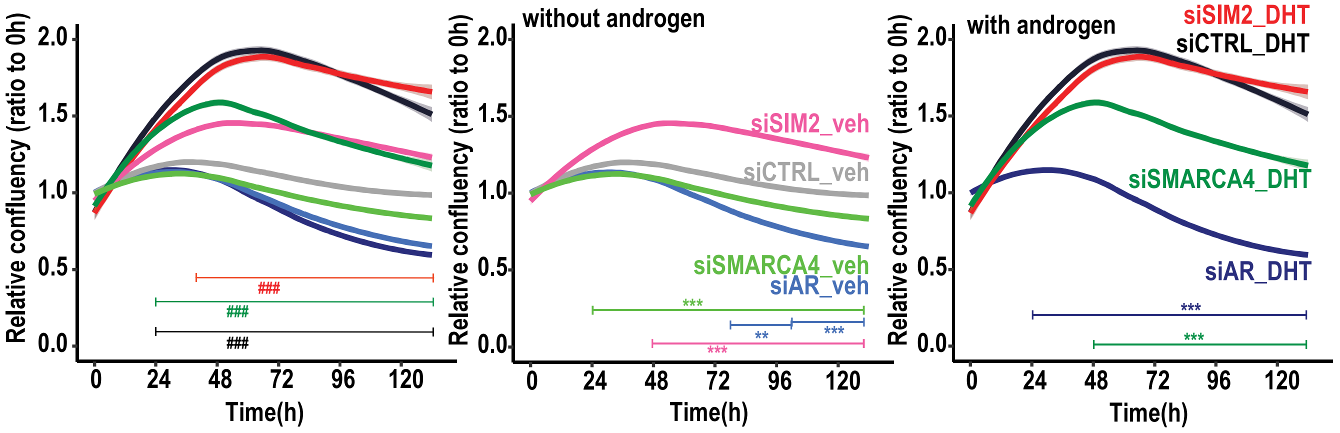

Supplementary Figure S10. Effect of siSMARCA4 and siSIM2 on the proliferation of LNCaP cells. LNCaP cells exposed to siCTRL, siAR, siSMARCA4 or siSIM2 as indicated were monitored by phase percentage with live cell imaging in Incucyte® in relation to the starting time point. Cells were exposed to 100 nM DHT or vehicle (veh, ethanol) as indicated at the start of the experiment. Significant changes in Two-way ANOVA and Bonferroni's multiple comparison posttests are indicated with asterisks, p-value **<0.01, ***<0.001 and significant hormone effect with ###<0.001. On the left, all cell confluency measurements in the same graph; in the middle, measurements in the absence of androgen; on the right, measurements in the presence of DHT. Line presents mean and the shadow around it indicates standard deviation from four biological replicates.

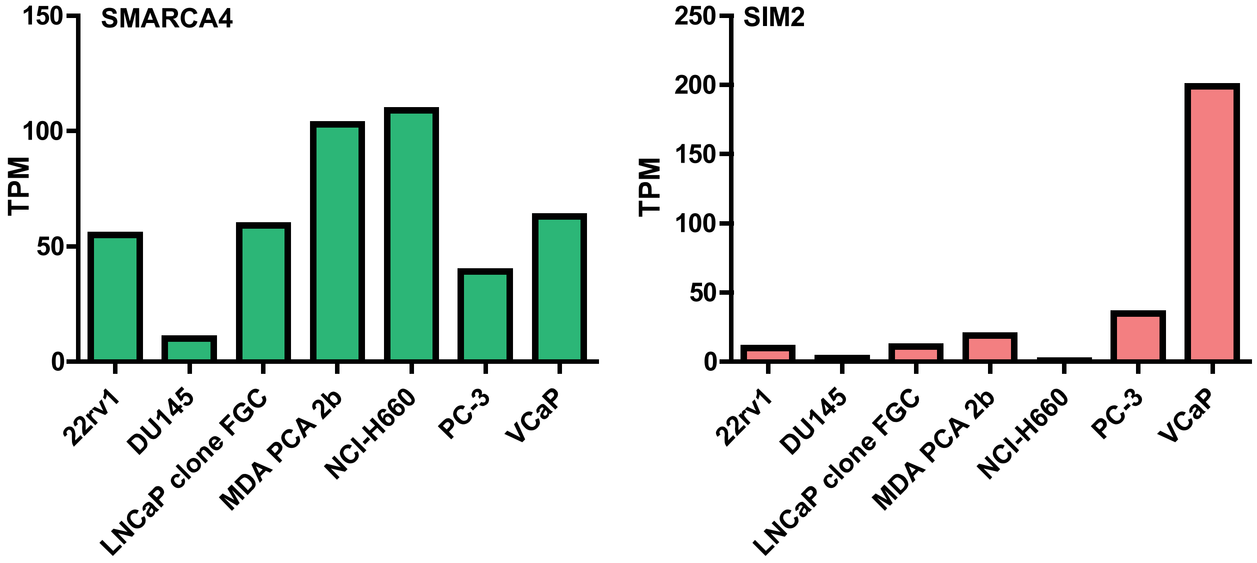

Supplementary Figure S11. Expression of SMARCA4 and SIM2 mRNA in various PCa cell lines. TPM-normalized mRNA expression levels of SMARCA4 mRNA and SIM2 mRNA from Cancer Cell Line Encyclopedia data [32].

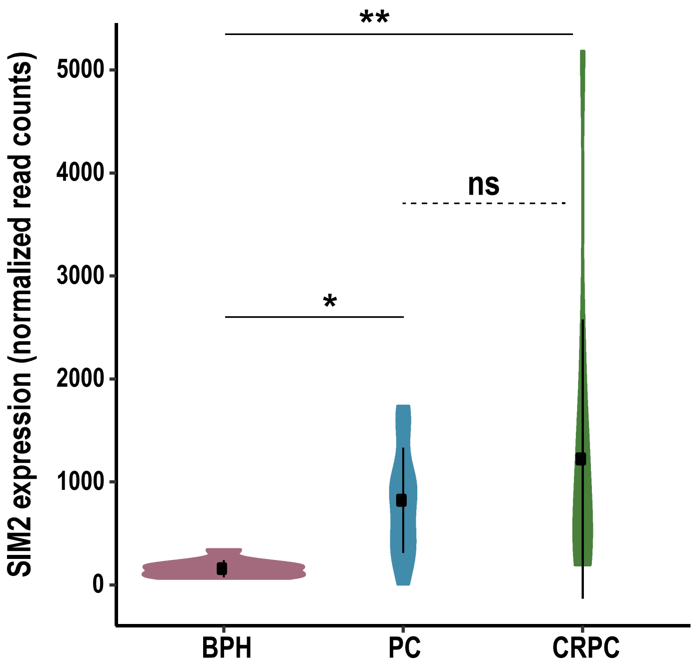

Supplementary Figure S12. Overexpression of SIM2 mRNA in clinical prostate cancer specimens. Expression of SIM2 in biopsy samples of prostate cancer (PC, n=30) and castration resistant prostate cancer (CRPC, n=13) compared to those of benign prostate hyperplasia (BPH, n=12) as measured by RNA-seq of a cohort described in Annala et al. [33]. Violins represents the distribution of samples, square dot presents mean and vertical line standard deviation from replicates. Significances were calculated by One-way ANOVA and Bonferroni's multiple comparison posttests comparing all columns, p-value *<0.05, and **<0.01.

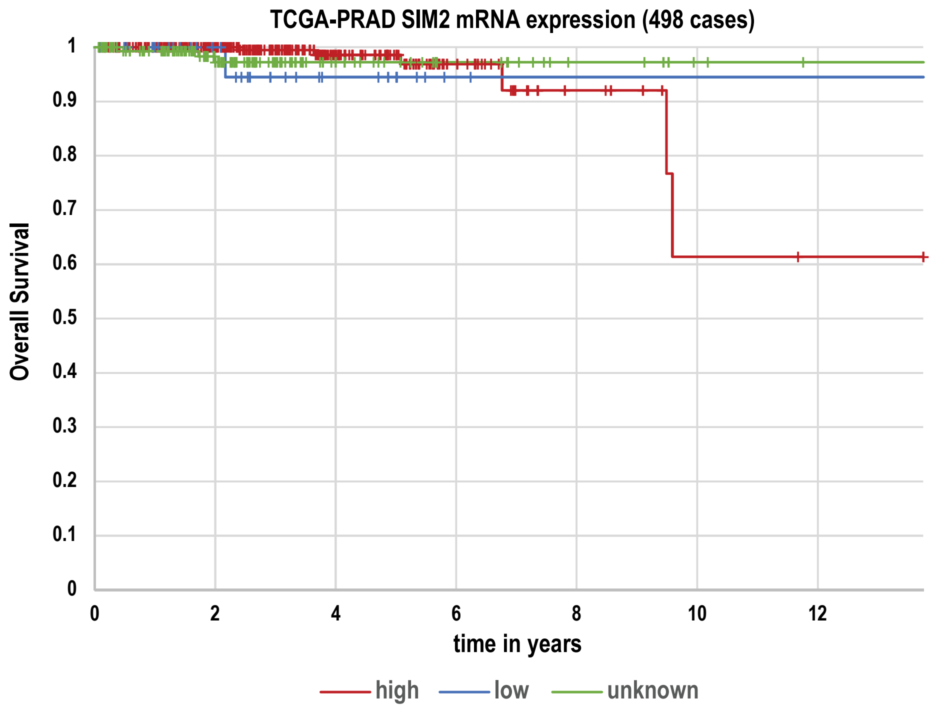

Supplementary Figure S13. Association of SIM2 mRNA expression with overall patient survival in TCGA prostate cancer cohort. Overall patient survival analysis of SIM2 mRNA expression (n = 498; expression high, z-score to normal samples > 1; expression low, z-score to normal samples ≤ 1). Survival estimate p-value for high to low comparison tested with Student's t-test, two-tailed distribution and assuming unequal variances is 0.0133.

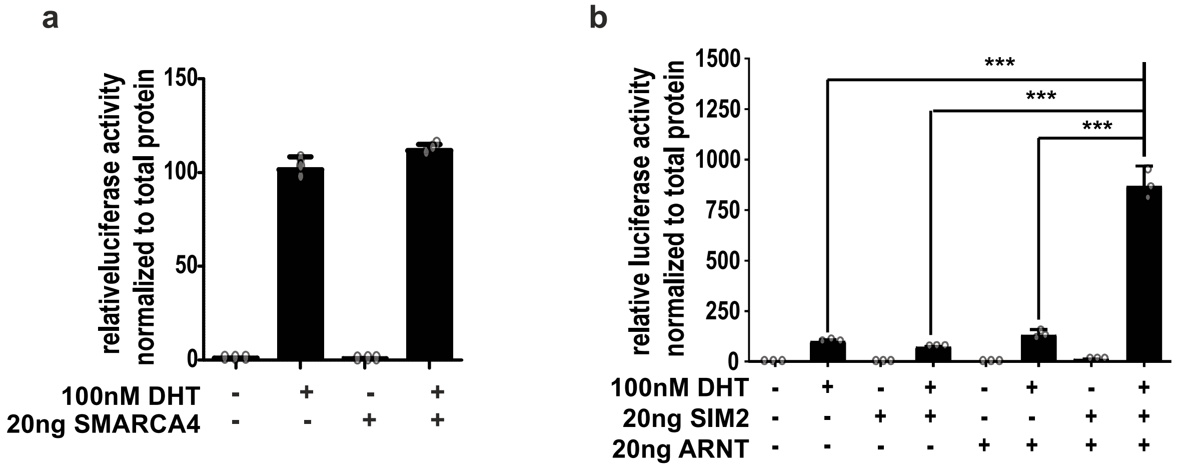

Supplementary Figure S14. Effect of SMARCA4, SIM2 and ARNT on an AR-dependent reporter gene in VCaP cells. Cells were co-transfected with pGL3-ARE2-TATA-LUC and SMARCA4 expression plasmid (**a**) or SIM2 and ARNT expression plasmids alone or together, as indicated (**b**) . Twenty-four h after transfection, cells were exposed to 100 nM DHT or vehicle for 17 h prior to measurement of luciferase activity (see Supplementary Methods for details). Luciferase activity was normalized to total protein amount. Columns presents mean ± standard deviation from three biological replicates. Significances were calculated by One-way ANOVA and Bonferroni's multiple comparison posttests comparing all columns,*** indicating p-value <0.001.

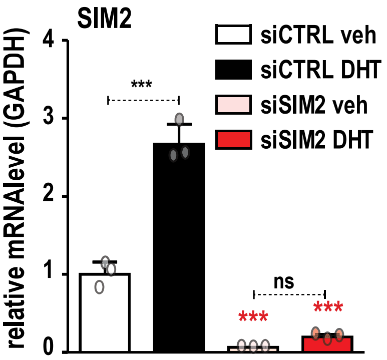

Supplementary Figure S15. Validation of SIM2 silencing by RT-qPCR. RT-qPCR analysis also reveals that the expression of SIM2 is significantly up-regulated by DHT in control (siCTRL treated) cells (***, p-value < 0.001). Columns presents mean ± standard deviation from three biological replicates. Interestingly, androgen ablation therapy decreases SIM2 expression in Expression Atlas dataset E-GEOD-48403 (log2(FC) = -1.5, p-value = $9.4523e^{-4}$, [34]), and the expression is also decreased after androgen deprivation therapy combined with docetaxel chemotherapy in dataset E-GEOD-51005 (log2(FC) = -1.4, p-value =$1.0064e^{-8}$, [35]).

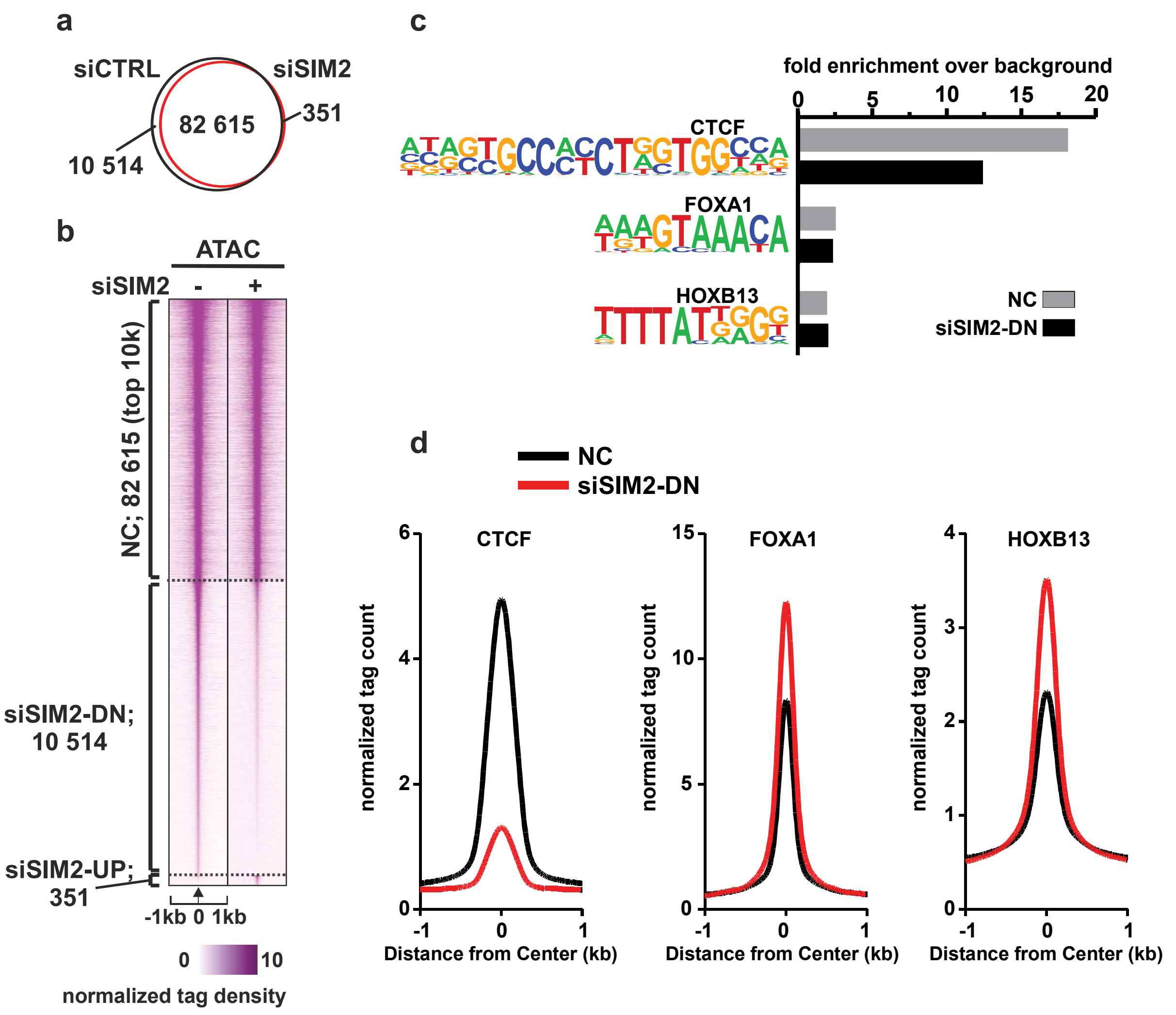

Supplementary Figure S16. Effect of SIM2 silencing on overall chromatin accessibillity in VCaP cells. a and **b** Chromatin accessibility sites as revealed by ATAC-seq in SIM2-silenced (siSIM2) and control (siCTRL) VCaP cells shown as Venn diagram (**a**) and as a heatmap of normalized tag counts (**b**). SIM2 silencing decreased the chromatin accessibility at 10 514 sites, whereas only at 351 sites, the chromatin accessibility was increased. Only top 10k of all chromatin accessibility sites that do not change by SIM2 are shown in the heatmap (NC). **c** Motif analysis of siSIM2-down-regulated chromatin accessibility sites revealed enrichment of motifs for CTCF, FOXA1 and HOXB13. For comparison, the enrichment of these motifs is shown for non-changed sites. **d** Binding of CTCF, FOXA1 or HOXB13 at NC and siSIM2-affected sites.

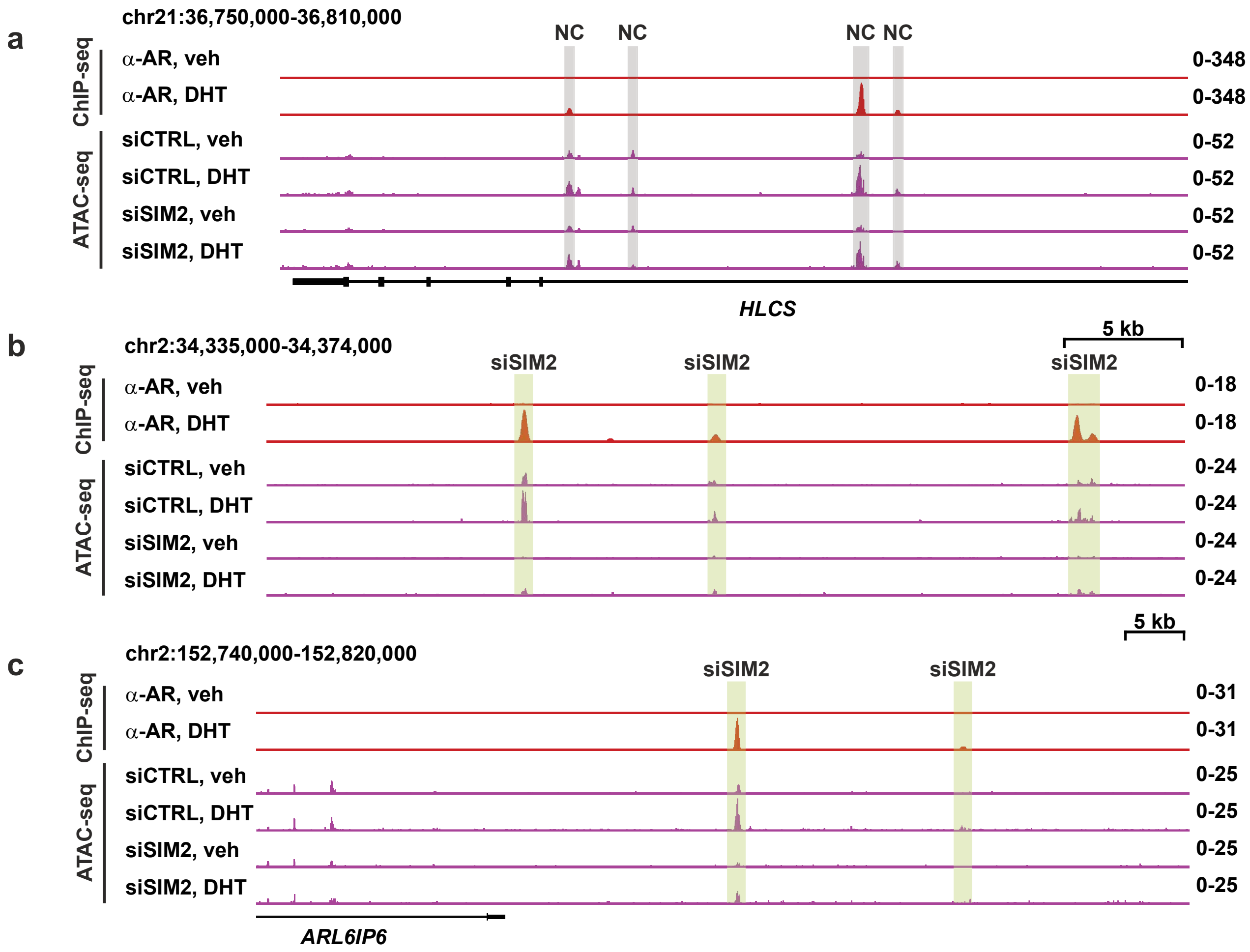

Supplementary Figure S17. Examples of AR-binding sites where SIM2 silencing alters chromatin accessibility. a AR-binding sites in *HLCS* locus shows non-changed (NC) sites. **b** Chr2:34,335,000-34,374,000 locus and **c** and *ARL6IP6* locus represent examples of diminished accessibility upon SIM2 silencing. On the right, scale of peak intensity for each track.

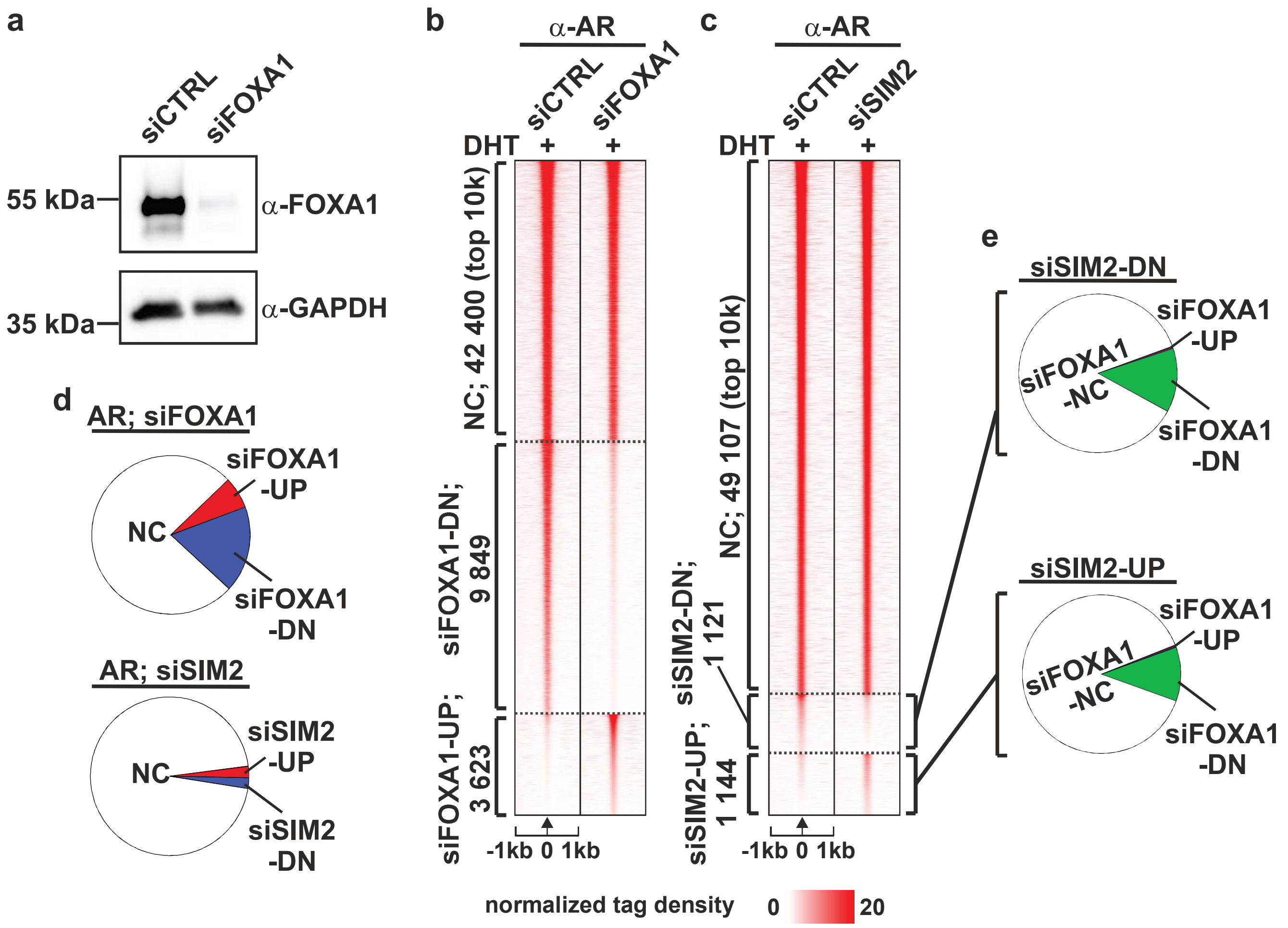

Supplementary Figure S18. Pioneering activity of SIM2 for AR is weaker than that of FOXA1. a Depletion of FOXA1 from VCaP cells was confirmed by western blotting with anti-FOXA1 antibody and anti-GAPDH antibody as a loading control. **b** AR-ChIP seq data are shown as heatmap of normalized tag densities upon FOXA1 depletion under DHT exposure and grouped by the effect of siFOXA1. Top10k of non-changed (NC) sites are shown together with total sites where AR binding is decreased (siFOXA1-DN) and increased (siFOXA1-UP) by siFOXA1. **c** AR-ChIP seq data are shown as heatmap of normalized tag densities upon SIM2 silencing under DHT exposure and grouped by siSIM2 effect. Top10k of non-changed (NC) sites are shown together with total sites where AR binding is decreased (siSIM2-DN) and increased (siSIM2-UP) by siSIM2. The amount of AR-binding sites down-regulated by siSIM2 (siSIM2-DN) represent ~10% of siFOXA1 down-regulated AR-binding sites (siFOXA1-DN), while the amount of up-regulated sites by siSIM2 (siSIM2-UP) or siFOXA1 (siFOXA1-UP) is more similar. **d** Pie charts showing the proportions of AR-binding site changes by siFOXA1 (upper pie) or siSIM2 (lower pie) of all AR-binding sites. **e** Pie charts showing the effect of siFOXA1 on AR binding on siSIM2-DN or siSIM2-UP groups.

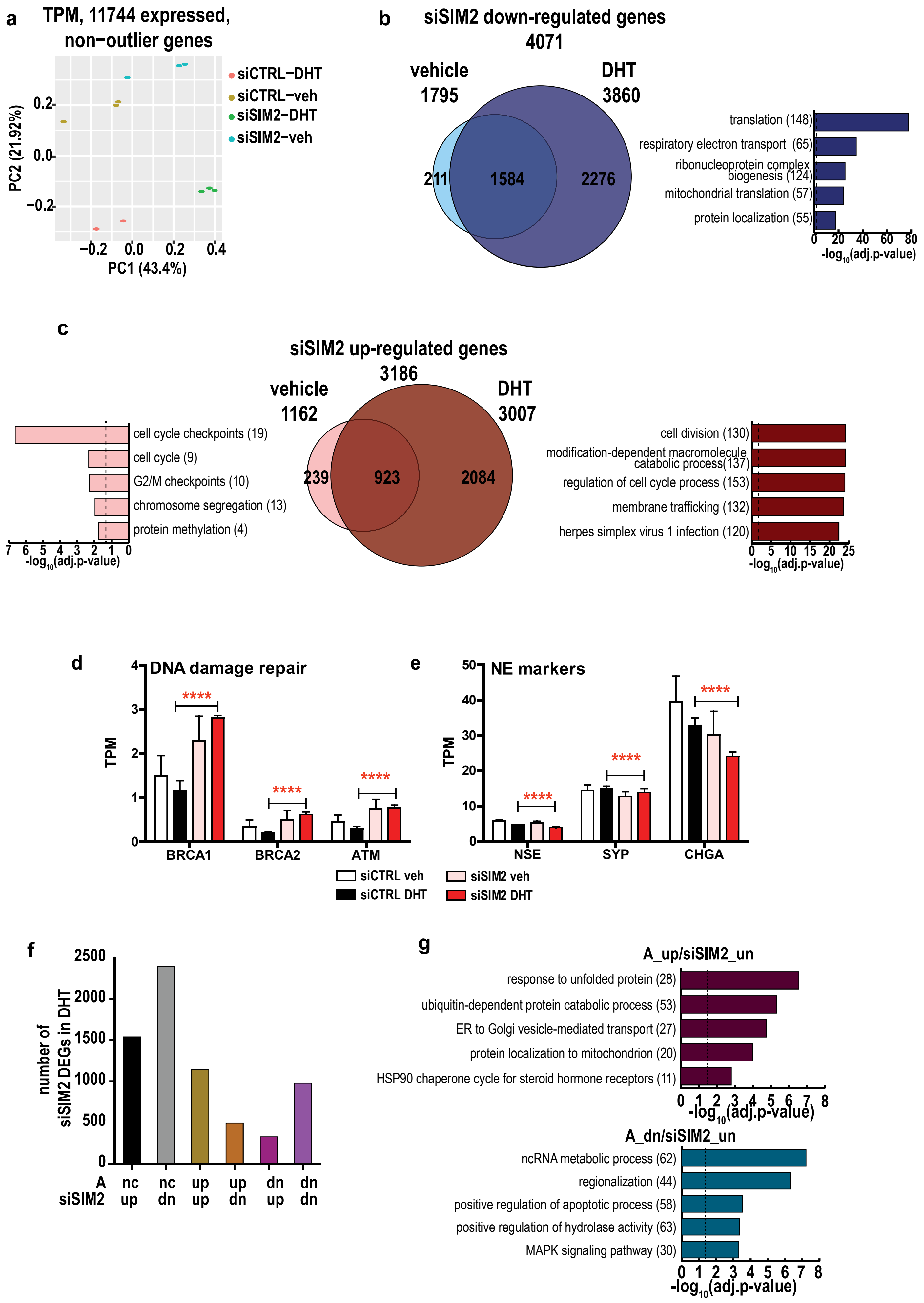
a
TPM, 11744 expressed, non−outlier genes
siCTRL−DHT
siCTRL−veh
siSIM2−DHT
siSIM2−veh
PC2 (21.92%)
PC1 (43.4%)
b
siSIM2 down-regulated genes
4071
vehicle
1795
DHT
3860
211
1584
2276
translation (148)
respiratory electron transport (65)
ribonucleoprotein complex biogenesis (124)
mitochondrial translation (57)
protein localization (55)
-log10(adj.p-value)
c
siSIM2 up-regulated genes
3186
vehicle
1162
DHT
3007
239
923
2084
cell cycle checkpoints (19)
cell cycle (9)
G2/M checkpoints (10)
chromosome segregation (13)
protein methylation (4)
cell division (130)
modification-dependent macromolecule catabolic process(137)
regulation of cell cycle process (153)
membrane trafficking (132)
herpes simplex virus 1 infection (120)
d
DNA damage repair
TPM
BRCA1
BRCA2
ATM
e
NE markers
NSE
SYP
CHGA
siCTRL veh
siCTRL DHT
siSIM2 veh
siSIM2 DHT
f
number of siSIM2 DEGs in DHT
A
siSIM2
g
A_up/siSIM2_un
response to unfolded protein (28)
ubiquitin-dependent protein catabolic process (53)
ER to Golgi vesicle-mediated transport (27)
protein localization to mitochondrion (20)
HSP90 chaperone cycle for steroid hormone receptors (11)
A_dn/siSIM2_un
ncRNA metabolic process (62)
regionalization (44)
positive regulation of apoptotic process (58)
positive regulation of hydrolase activity (63)
MAPK signaling pathway (30)

Supplementary Figure S19. Effect of SIM2 silencing on gene expression in VCaP cells as assessed by RNA-seq. a Principal component analysis: PC1 differentiates samples by siRNA (siCTRL, siSIM2) and PC2 by androgen exposure (vehicle, veh; androgen, DHT). **b** Venn diagram shows overlap of siSIM2 down-regulated genes in the presence of vehicle or DHT. Dark blue bar graph presents the enriched pathways in Metascape analysis for the genes down-regulated by siSIM2 in the presence of DHT (2276 genes). The group of 211 genes down-regulated by siSIM2 in the presence of vehicle does not enrich in any pathway. **c** Venn diagram shows the overlap of siSIM2-up-regulated genes in vehicle (light red) or DHT treatment (dark red) with respective top five enriched pathways in bar graphs. **d** SIM2 silencing enhances the expression of *BRCA1, BRCA2* and *ATM* and blunts the inhibitory effect of DHT on their expression. **e** Expression of neuroendocrine marker genes *NSE, SYP* and *CHGA* is altered by SIM2 silencing. Columns presents mean ± standard deviation from three biological replicates. Asterisks indicate significant difference between siCTRL and siSIM2 in red, adj.p-value ****< 0.0001 by DESeq2 analysis in HOMER. **f** Bar graph illustrates the portions of all differentially regulated genes and division by the effect of siSIM2 and androgen. **g** Top five enriched pathways in androgen up- and down-regulated genes unchanged by SIM2 silencing (clusters A_up/siSIM2_un and A_dn/siSIM2_un in Figure 8). Dashed lines in pathway bar graphs indicate the adjusted p-value 0.05.

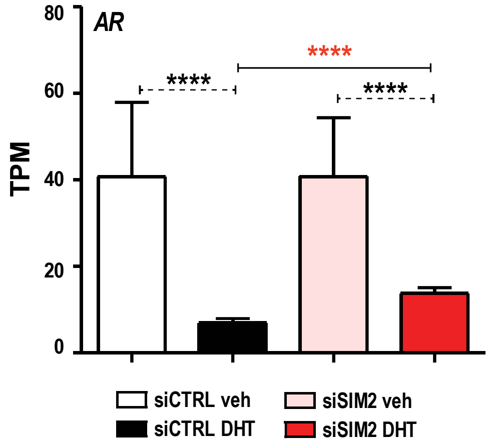

Supplementary Figure S20. The effect of SIM2 silencing on the expression of AR. Based on RNA-seq data, SIM2 silencing blunts the inhibitory effect of DHT on AR mRNA expression. Columns presents mean ± standard deviation from three biological replicates. Asterisks in red indicate significant difference between siCTRL and siSIM2 and the black ones indicate significant hormone effect, adj.p-value ****< 0.0001 by DESeq2 analysis in HOMER.

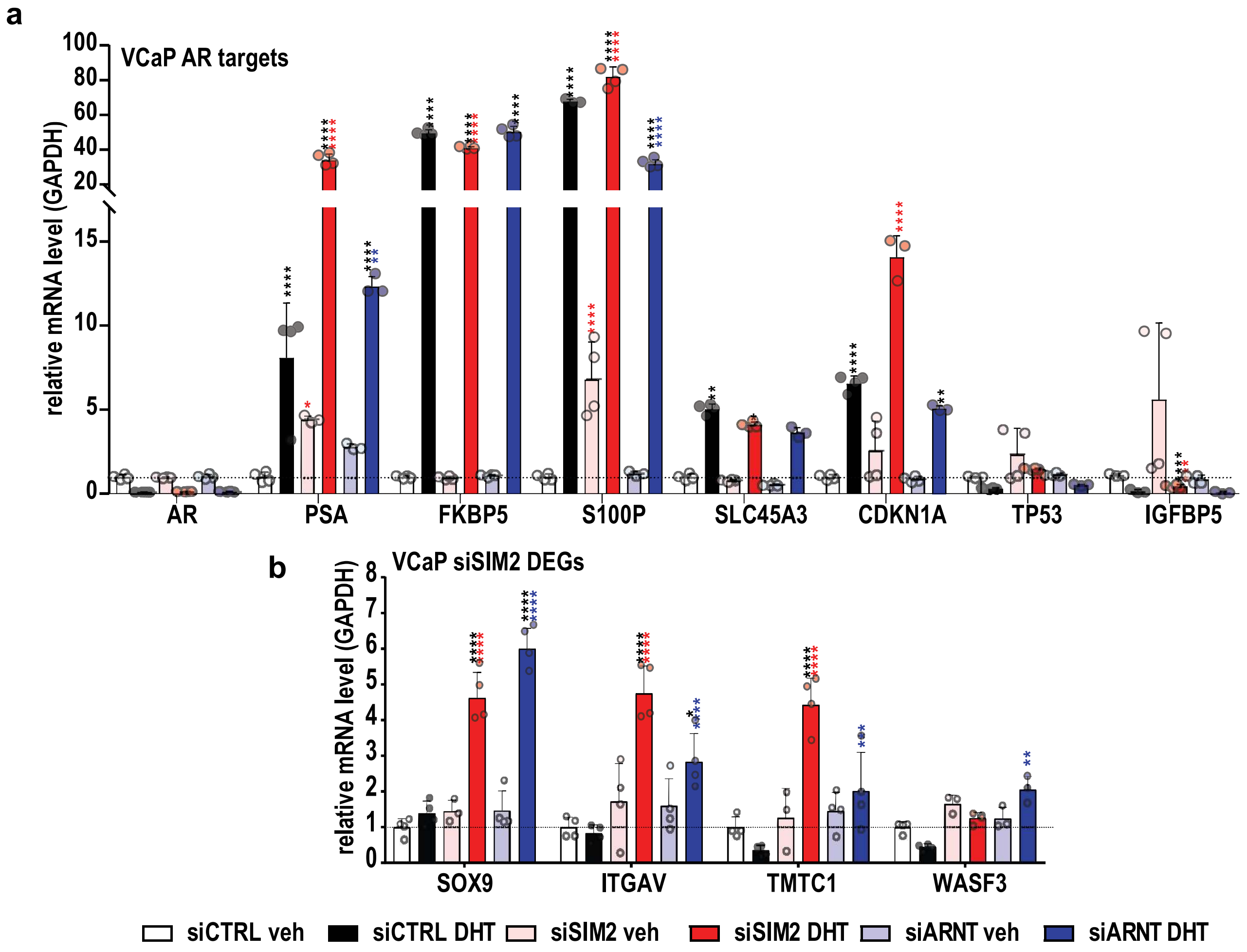

Supplementary Figure S21. The effect of SIM2 or ARNT silencing on the expression selected AR target genes and siSIM2-affected DEGs in VCaP cells. a Silencing of ARNT essentially recapitulates the effect of SIM2 silencing on the expression of indicated AR target genes as analysed by RT-qPCR. **b** The effect of ARNT silencing on selected DEGs resulting from SIM2 silencing. Columns presents mean ± standard deviation from four biological replicates. All levels are normalized to GAPDH, and with each gene to its siCTRL- and vehicle (veh, EtOH)-treated control (dashed line). Significant differences between siCTRL and siSIM2 (in red) or siARNT (in blue) are shown by asterisks with significant hormone effect by black asterisks on top of the DHT columns. Significance is calculated by Two-way ANOVA and Tukey's multiple comparison posttests comparing all columns, p-value *<0.05, **<0.01**, ***<0.001 and ****<0.0001.

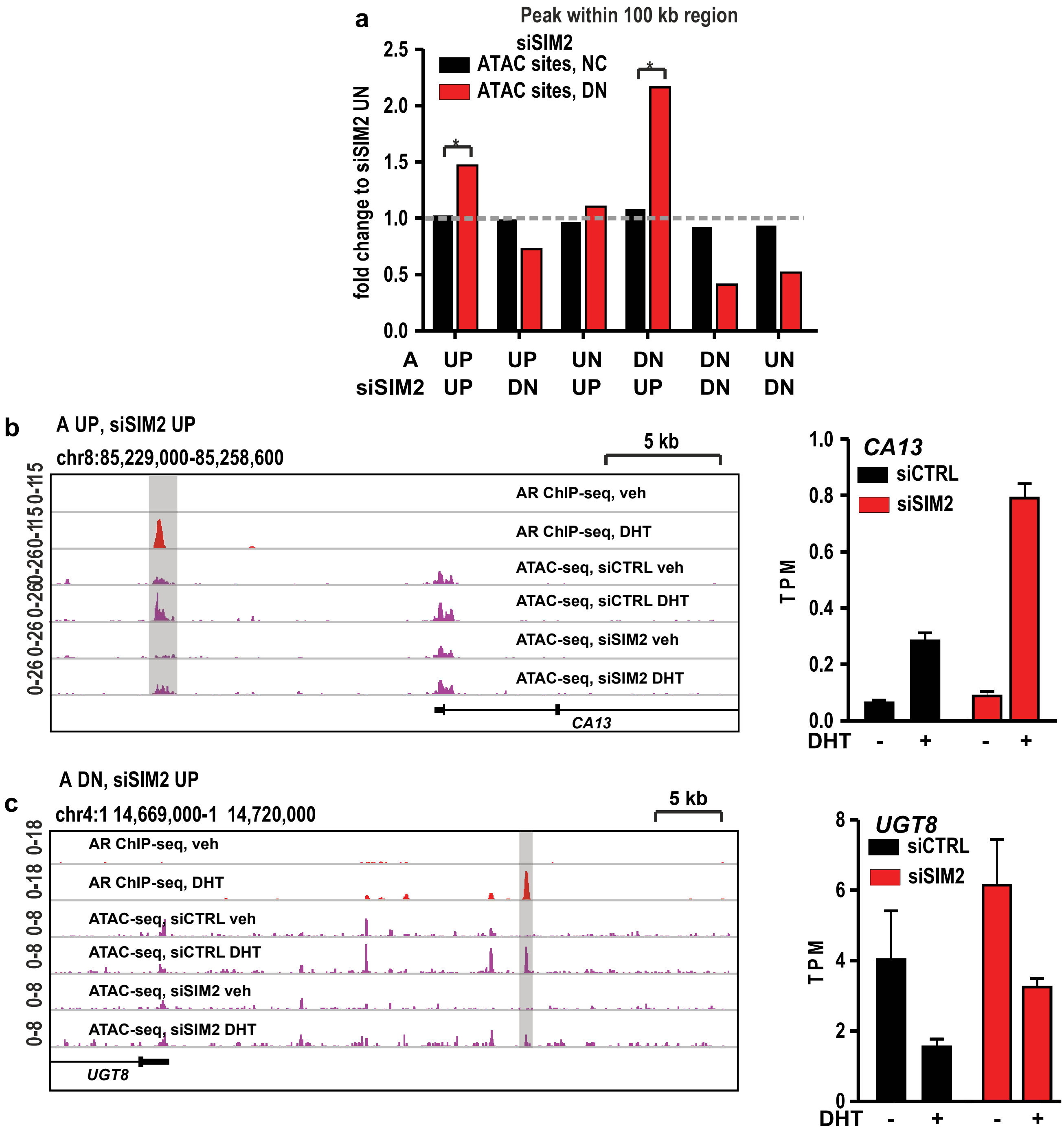

Supplementary Figure S22. Association of siSIM2-enhanced and androgen-regulated DEGs with siSIM2-affected ATAC-seq sites. a Association of DEGs in siSIM2 treatment (Figure 8, Supplementary Figure S21) with ATAC-seq peaks (Figure 6) with the search criteria of peak within 100 kb from transcription start site and normalization to androgen-regulated genes whose expression does not change with siSIM2 upon DHT exposure. Dashed horizontal line defines the background association. **b** and **c** Genome browser tracks of genes CA13 and UTG8 and their associated ATAC peaks are shown as examples. On the right, bar graphs show the corresponding changes in gene expression. Columns presents mean ± standard deviation from three biological replicates.

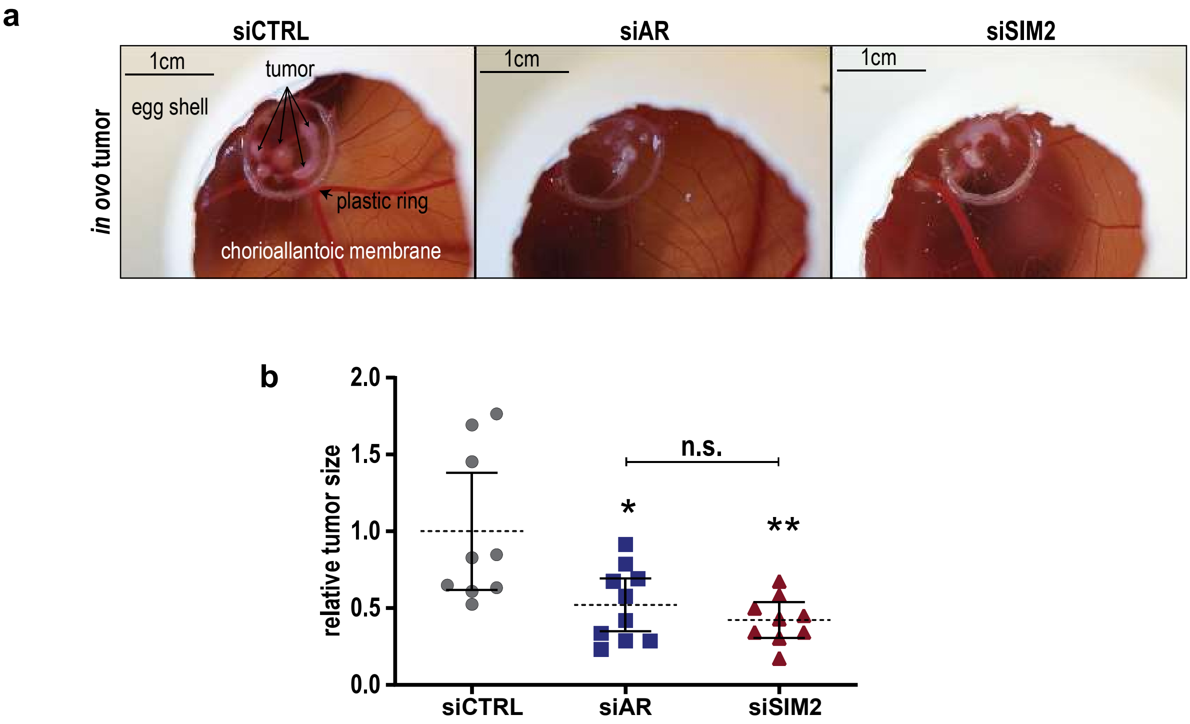

Supplementary Figure S23. SIM2 silencing decreases tumor volume similarly as AR silencing in chorioallantoic membrane assay. a Representative figures from *in ovo* tumors in chorioallantoic membrane assay. VCaP cells were reverse transfected with specific siRNAs for SIM2, AR or with non-targeting control (siCTRL) in growth medium for five days before inoculation onto eggs on embryo development day (EDD) 8. siRNA-treated cells were applied inside a plastic ring on allantoic vein and incubated for four days. EDD12 tumors were photographed *in ovo,* and tumor size was evaluated from 9 to 10 eggs per silencing condition. VCaP cells tend to form multinodular nests instead of large solitary tumors. **b** Quantification of *in ovo* tumor areas normalized to ring area in chorioallantoic membrane assay (see Methods for details). Whiskers indicate the 95% confidence interval and dashed line mean. Significances are indicated as asterisks *p-value<0.05 and **p-value<0.01 in One-Way ANOVA and Bonferroni post-test. n=9-10.

Supplementary references

1 Paakinaho V, Kaikkonen S, Makkonen H, Benes V and Palvimo JJ (2014) SUMOylation regulates the chromatin occupancy and anti-proliferative gene programs of glucocorticoid receptor. Nucleic Acids Res 42:1575-1592

2 Karvonen U, Kallio PJ, Janne OA and Palvimo JJ (1997) Interaction of androgen receptors with androgen response element in intact cells. Roles of amino- and carboxyl-terminal regions and the ligand. J Biol Chem 272:15973-15979

3 Rafiee MR, Girardot C, Sigismondo G and Krijgsveld J (2016) Expanding the Circuitry of Pluripotency by Selective Isolation of Chromatin-Associated Proteins. Mol Cell 64:624-635

4 Rafiee MR, Sigismondo G, Kalxdorf M, Forster L, Brugger B, Bethune J and Krijgsveld J (2020) Protease-resistant streptavidin for interaction proteomics. Mol Syst Biol 16:e9370

5 Hughes CS, Foehr S, Garfield DA, Furlong EE, Steinmetz LM and Krijgsveld J (2014) Ultrasensitive proteome analysis using paramagnetic bead technology. Mol Syst Biol 10:757

6 Hughes CS, Sorensen PH and Morin GB (2019) A Standardized and Reproducible Proteomics Protocol for Bottom-Up Quantitative Analysis of Protein Samples Using SP3 and Mass Spectrometry. Methods Mol Biol 1959:65-87

7 Edie S, Zaghloul NA, Leitch CC, Klinedinst DK, Lebron J, Thole JF, McCallion AS, Katsanis N and Reeves RH (2018) Survey of Human Chromosome 21 Gene Expression Effects on Early Development in Danio rerio. G3 (Bethesda) 8:2215-2223

8 Chen W, Hill H, Christie A, Kim MS, Holloman E, Pavia-Jimenez A, Homayoun F, Ma Y, Patel N, Yell P, Hao G, Yousuf Q, Joyce A, Pedrosa I, Geiger H, Zhang H, Chang J, Gardner KH, Bruick RK, Reeves C, Hwang TH, Courtney K, Frenkel E, Sun X, Zojwalla N, Wong T, Rizzi JP, Wallace EM, Josey JA, Xie Y, Xie XJ, Kapur P, McKay RM and Brugarolas J (2016) Targeting renal cell carcinoma with a HIF-2 antagonist. Nature 539:112-117

9 Toropainen S, Malinen M, Kaikkonen S, Rytinki M, Jaaskelainen T, Sahu B, Janne OA and Palvimo JJ (2015) SUMO ligase PIAS1 functions as a target gene selective androgen receptor coregulator on prostate cancer cell chromatin. Nucleic Acids Res 43:848-861

10 Makkonen H, Jaaskelainen T, Rytinki MM and Palvimo JJ (2011) Analysis of androgen receptor activity by reporter gene assays. Methods Mol Biol 776:71-80

11 Buenrostro JD, Wu B, Chang HY and Greenleaf WJ (2015) ATAC-seq: A Method for Assaying Chromatin Accessibility Genome-Wide. Curr Protoc Mol Biol 109:21.29.1-21.29.9

12 Paakinaho V, Johnson TA, Presman DM and Hager GL (2019) Glucocorticoid receptor quaternary structure drives chromatin occupancy and transcriptional outcome. Genome Res 29:1223-1234

13 Buenrostro JD, Giresi PG, Zaba LC, Chang HY and Greenleaf WJ (2013) Transposition of native chromatin for fast and sensitive epigenomic profiling of open chromatin, DNA-binding proteins and nucleosome position. Nat Methods 10:1213-1218

14 Harkonen K, Oikari S, Kyykallio H, Capra J, Hakkola S, Ketola K, Thanigai Arasu U, Daaboul G, Malloy A, Oliveira C, Jokelainen O, Sironen R, Hartikainen JM and Rilla K (2019) CD44s Assembles Hyaluronan Coat on Filopodia and Extracellular Vesicles and Induces Tumorigenicity of MKN74 Gastric Carcinoma Cells. Cells 8:10.3390/cells8030276

15 Schneider CA, Rasband WS and Eliceiri KW (2012) NIH Image to ImageJ: 25 years of image analysis. Nat Methods 9:671-675

16 Cox J and Mann M (2008) MaxQuant enables high peptide identification rates, individualized p.p.b.-range mass accuracies and proteome-wide protein quantification. Nat Biotechnol 26:1367-1372

17 Tyanova S, Temu T and Cox J (2016) The MaxQuant computational platform for mass spectrometry-based shotgun proteomics. Nat Protoc 11:2301-2319

18 Perez-Riverol Y, Csordas A, Bai J, Bernal-Llinares M, Hewapathirana S, Kundu DJ, Inuganti A, Griss J, Mayer G, Eisenacher M, Perez E, Uszkoreit J, Pfeuffer J, Sachsenberg T, Yilmaz S, Tiwary S, Cox J, Audain E, Walzer M, Jarnuczak AF, Ternent T, Brazma A and Vizcaino JA (2019) The PRIDE database and related tools and resources in 2019: improving support for quantification data. Nucleic Acids Res 47:D442-D450

19 Ritchie ME, Phipson B, Wu D, Hu Y, Law CW, Shi W and Smyth GK (2015) limma powers differential expression analyses for RNA-sequencing and microarray studies. Nucleic Acids Res 43:e47

20 Langmead B and Salzberg SL (2012) Fast gapped-read alignment with Bowtie 2. Nat Methods 9:357-359

21 Heinz S, Benner C, Spann N, Bertolino E, Lin YC, Laslo P, Cheng JX, Murre C, Singh H and Glass CK (2010) Simple combinations of lineage-determining transcription factors prime cis-regulatory elements required for macrophage and B cell identities. Mol Cell 38:576-589

22 Dobin A, Davis CA, Schlesinger F, Drenkow J, Zaleski C, Jha S, Batut P, Chaisson M and Gingeras TR (2013) STAR: ultrafast universal RNA-seq aligner. Bioinformatics 29:15-21

23 Zhou Y, Zhou B, Pache L, Chang M, Khodabakhshi AH, Tanaseichuk O, Benner C and Chanda SK (2019) Metascape provides a biologist-oriented resource for the analysis of systems-level datasets. Nat Commun 10:1523-019-09234-6

24 Sandoval GJ, Pulice JL, Pakula H, Schenone M, Takeda DY, Pop M, Boulay G, Williamson KE, McBride MJ, Pan J, St Pierre R, Hartman E, Garraway LA, Carr SA, Rivera MN, Li Z, Ronco L, Hahn WC and Kadoch C (2018) Binding of TMPRSS2-ERG to BAF Chromatin Remodeling Complexes Mediates Prostate Oncogenesis. Mol Cell 71:554-566.e7

25 Davis CA, Hitz BC, Sloan CA, Chan ET, Davidson JM, Gabdank I, Hilton JA, Jain K, Baymuradov UK, Narayanan AK, Onate KC, Graham K, Miyasato SR, Dreszer TR, Strattan JS, Jolanki O, Tanaka FY and Cherry JM (2018) The Encyclopedia of DNA elements (ENCODE): data portal update. Nucleic Acids Res 46:D794-D801

26 Valdes-Mora F, Gould CM, Colino-Sanguino Y, Qu W, Song JZ, Taylor KM, Buske FA, Statham AL, Nair SS, Armstrong NJ, Kench JG, Lee KML, Horvath LG, Qiu M, Ilinykh A, Yeo-Teh NS, Gallego-Ortega D, Stirzaker C and Clark SJ (2017) Acetylated histone variant H2A.Z is involved in the activation of neo-enhancers in prostate cancer. Nat Commun 8:1346-017-01393-8

27 Sharma NL, Massie CE, Butter F, Mann M, Bon H, Ramos-Montoya A, Menon S, Stark R, Lamb AD, Scott HE, Warren AY, Neal DE and Mills IG (2014) The ETS family member GABPalpha modulates androgen receptor signalling and mediates an aggressive phenotype in prostate cancer. Nucleic Acids Res 42:6256-6269

28 Kron KJ, Murison A, Zhou S, Huang V, Yamaguchi TN, Shiah YJ, Fraser M, van der Kwast T, Boutros PC, Bristow RG and Lupien M (2017) TMPRSS2-ERG fusion co-opts master transcription factors and activates NOTCH signaling in primary prostate cancer. Nat Genet 49:1336-1345

29 Toropainen S, Niskanen EA, Malinen M, Sutinen P, Kaikkonen MU and Palvimo JJ (2016) Global analysis of transcription in castration-resistant prostate cancer cells uncovers active enhancers and direct androgen receptor targets. Sci Rep 6:33510

30 Stelloo S, Nevedomskaya E, Kim Y, Hoekman L, Bleijerveld OB, Mirza T, Wessels LFA, van Weerden WM, Altelaar AFM, Bergman AM and Zwart W (2018) Endogenous androgen receptor proteomic profiling reveals genomic subcomplex involved in prostate tumorigenesis. Oncogene 37:313-322

31 Paltoglou S, Das R, Townley SL, Hickey TE, Tarulli GA, Coutinho I, Fernandes R, Hanson AR, Denis I, Carroll JS, Dehm SM, Raj GV, Plymate SR, Tilley WD and Selth LA (2017) Novel Androgen Receptor Coregulator GRHL2 Exerts Both Oncogenic and Antimetastatic Functions in Prostate Cancer. Cancer Res 77:3417-3430

32 Barretina J, Caponigro G, Stransky N, Venkatesan K, Margolin AA, Kim S, Wilson CJ, Lehár J, Kryukov GV, Sonkin D, Reddy A, Liu M, Murray L, Berger MF, Monahan JE, Morais P, Meltzer J, Korejwa A, Jané-Valbuena J, Mapa FA, Thibault J, Bric-Furlong E, Raman P, Shipway A, Engels IH, Cheng J, Yu GK, Yu J, Aspesi P Jr, de Silva M, Jagtap K, Jones MD, Wang L, Hatton C, Palescandolo E, Gupta S, Mahan S, Sougnez C, Onofrio RC, Liefeld T, MacConaill L, Winckler W, Reich M, Li N, Mesirov JP, Gabriel SB, Getz G, Ardlie K, Chan V, Myer VE, Weber BL, Porter J, Warmuth M, Finan P, Harris JL, Meyerson M, Golub TR, Morrissey MP, Sellers WR, Schlegel R, Garraway LA. The Cancer Cell Line Encyclopedia enables predictive modelling of anticancer drug sensitivity. Nature. 2012 Mar 28;483(7391):603-7.

33 Annala M, Kivinummi K, Tuominen J, Karakurt S, Granberg K, Latonen L, Ylipää A, Sjöblom L, Ruusuvuori P, Saramäki O, Kaukoniemi KM, Yli-Harja O, Vessella RL, Tammela TL, Zhang W, Visakorpi T, Nykter M. Recurrent SKIL-activating rearrangements in ETS-negative prostate cancer. Oncotarget. 2015 Mar 20;6(8):6235-50.

34 Rajan P, Sudbery IM, Villasevil ME, Mui E, Fleming J, Davis M, Ahmad I, Edwards J, Sansom OJ, Sims D, Ponting CP, Heger A, McMenemin RM, Pedley ID, Leung HY. Next-generation sequencing of advanced prostate cancer treated with androgen-deprivation therapy. Eur Urol. 2014 Jul;66(1):32-9. doi: 10.1016/j.eururo.2013.08.011. Epub 2013 Aug 14. .

35 Rajan P, Stockley J, Sudbery IM, Fleming JT, Hedley A, Kalna G, Sims D, Ponting CP, Heger A, Robson CN, McMenemin RM, Pedley ID, Leung HY. Identification of a candidate prognostic gene signature by transcriptome analysis of matched pre- and post-treatment prostatic biopsies from patients with advanced prostate cancer. BMC Cancer. 2014 Dec 18;14:977.
